# A gene expression signature of retinoblastoma loss-of-function is a predictive biomarker of resistance to palbociclib in breast cancer cell lines and is prognostic in patients with ER positive early breast cancer

**DOI:** 10.18632/oncotarget.12010

**Published:** 2016-09-13

**Authors:** Luca Malorni, Silvano Piazza, Yari Ciani, Cristina Guarducci, Martina Bonechi, Chiara Biagioni, Christopher D. Hart, Roberto Verardo, Angelo Di Leo, Ilenia Migliaccio

**Affiliations:** ^1^ “Sandro Pitigliani” Translational Research Unit, Hospital of Prato-AUSL Toscana Centro, Istituto Toscano Tumori, Prato, Italy; ^2^ “Sandro Pitigliani” Medical Oncology Department, Hospital of Prato-AUSL Toscana Centro, Istituto Toscano Tumori, Prato, Italy; ^3^ Laboratorio Nazionale CIB (LNCIB), Area Science Park, and Functional Genomics & Bioinformatics Units, Trieste, Italy; ^4^ Bioinformatics Core Facility, Centre for Integrative Biology (CIBIO), University of Trento, Trento, Italy

**Keywords:** breast cancer, CDK4/6 inhibitors, retinoblastoma loss of functions

## Abstract

Palbociclib is a CDK4/6 inhibitor that received FDA approval for treatment of hormone receptor positive (HR+) HER2 negative (HER2neg) advanced breast cancer. To better personalize patients treatment it is critical to identify subgroups that would mostly benefit from it. We hypothesize that complex alterations of the Retinoblastoma (Rb) pathway might be implicated in resistance to CDK4/6 inhibitors and aim to investigate whether signatures of Rb loss-of-function would identify breast cancer cell lines resistant to palbociclib. We established a gene expression signature of Rb loss-of-function (RBsig) by identifying genes correlated with E2F1 and E2F2 expression in breast cancers within The Cancer Genome Atlas. We assessed the RBsig prognostic role in the METABRIC and in a comprehensive breast cancer meta-dataset. Finally, we analyzed whether RBsig would discriminate palbociclib-sensitive and -resistant breast cancer cells in a large RNA sequencing-based dataset. The RBsig was associated with RB1 genetic status in all tumors (*p* <7e-32) and in luminal or basal subtypes (*p* < 7e-11 and *p* < 0.002, respectively). The RBsig was prognostic in the METABRIC dataset (discovery: HR = 1.93 [1.5-2.4] *p* = 1.4e-08; validation: HR = 2.01 [1.6-2.5] *p* = 1.3e-09). Untreated and endocrine treated patients with estrogen receptor positive breast cancer expressing high RBsig had significantly worse recurrence free survival compared to those with low RBsig (HR = 2.37 [1.8 − 3.2] *p* = 1.87e−08 and HR = 2.62 [1.9− 3.5] *p* = 8.6e−11, respectively). The RBsig was able to identify palbociclib resistant and sensitive breast cancer cells (ROC AUC = 0,7778). Signatures of RB loss might be helpful in personalizing treatment of patients with HR+/HER2neg breast cancer. Further validation in patients receiving palbociclib is warranted.

## INTRODUCTION

Cell cycle related genes and proteins are frequently deregulated in breast cancer leading to uncontrolled cell proliferation, a hallmark of cancer [1]. The retinoblastoma susceptibility gene product (Rb) is a key regulator of cell cycle progression [2]. Together with other Rb family members (such as p107 and p130) Rb is phosphorylated by CyclinD1-Cyclin Dependent Kinases 4 and 6 (CDK4/6) and other cyclin-CDKs complexes inducing the release of transcriptional factors of the E2F family and the consequent transcription of genes required for S-phase entry [2]. This mechanism is tightly regulated and negatively modulated by proteins such as p16^ink4a^ [2]. In breast cancer, molecular alterations involving the CyclinD-CDK4/6-Rb pathway frequently occur [3] and strategies to target this pathway have recently been proven to be effective in patients with advanced breast cancer, particularly those with hormone receptor positive (HR+), HER2 negative (HER2neg) tumors [4].

Three different CDK4/6 inhibitors (palbociclib, abemaciclib and ribociclib) are in clinical development for the treatment of patients with HR+/HER2neg breast cancer, mostly in combination with endocrine therapy [5]. Palbociclib, a CDK4/6 inhibitor, has received accelerated approval in the U.S by the Food and Drug Administration (FDA) for the first line treatment of HR+/HER2neg advanced breast cancer in combination with the hormonal treatment letrozole [6] and, more recently, for the treatment of endocrine pre-treated patients in combination with the anti-estrogen fulvestrant, given the results of three pivotal randomized clinical trials, the PALOMA-1, PALOMA-2 and PALOMA-3 [7-10]. PALOMA-1 and PALOMA-2 are phase II and phase III randomized trials, respectively, of palbociclib in combination with letrozole versus letrozole alone for previously untreated patients in the metastatic setting [8, 10]; PALOMA-3 is a phase III randomized trial of palbociclib and fulvestrant versus placebo and fulvestrant for the treatment of patients relapsed on or progressed to a previous line of hormonal therapy [7, 9]. These trials clearly demonstrated the superiority of the combination over hormonal treatment alone in both hormone therapy-untreated and -pretreated populations [7-10]. Abemaciclib, another CDK4/6 inhibitor, has recently been granted FDA breakthrough therapy designation for patients with refractory HR+ metastatic breast cancer based on encouraging results from a phase I study in which single-agent abemaciclib demonstrated a clinical benefit rate of 61.1% in patients with heavily pre-treated metastatic breast cancer [11]. Whilst CDK4/6 inhibitors are well tolerated, they are not devoid of side-effects [7, 8, 11]; additionally, the financial costs of treatment are relevant. Clearly, understanding which subgroup of patients is not likely to benefit from CDK4/6 inhibitors is of critical importance.

Preclinical studies demonstrated that alterations in the CyclinD-CDK4/6-Rb pathway may have a role in primary resistance to palbociclib [12-15]. In particular, genetic loss of RB1 has been linked to resistance while high levels of Rb and CCND1 (cyclin D1) and low levels of CDKN2A (p16) have been associated to sensitivity to this compound [12, 13, 15]. However not all tumors that retain Rb are sensitive to CDK4/6 inhibitors and screening tumors for Rb or p16 expression and CCND1 amplification in phase II trials with palbociclib has not proven to be a successful strategy in discriminating responsive versus non-responsive tumors [8, 16]. More complex alterations of the pathway are likely to be implicated in resistance to palbociclib and these alterations may not be captured by single biomarkers. Gene-expression studies allow the simultaneous evaluation of the whole transcriptome permitting to derive information about specific pathways and cellular functions of interest. Gene-expression signatures such as the Oncotype DX®, Mammaprint® and others have already established as clinically valuable “biomarkers” in breast cancer. Gene-expression signatures focusing on inactivation of the Rb pathway have been developed and characterized in breast cancer patients datasets [17-23]. However, the correlation of such signatures with response to CDK4/6 inhibitors has never been explored. We hypothesized that a gene expression signature of functional loss of Rb might be explored as a potential novel biomarker of response to CDK4/6 inhibitors.

In this study, using a novel approach, we derived a new signature of Rb loss-of-function (RBsig) with the specific aim of testing whether this might help in discriminating between palbociclib resistant versus sensitive breast cancer cell lines. We also explored the prognostic role of our RBsig in well annotated datasets of patients with early breast cancer.

## RESULTS AND DISCUSSION

### RBsig: Creation of the signature, functional analysis and correlation with RB1 mutational status and molecular subtypes

**Figure 1:**
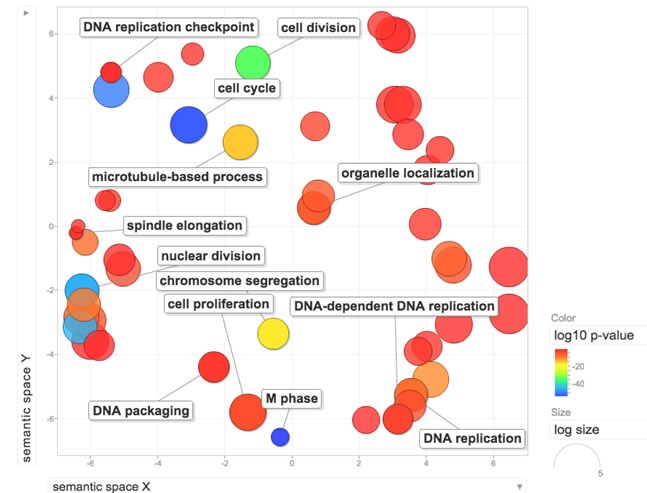
Functional analysis of the RBsig Enriched GO terms within the RBsig were plotted in a bi-dimensional space using a clustering algorithm that relies on semantic similarity measures using REVIGO tool [33].

To test our hypothesis that a signature of functional Rb loss would be predictive of palbociclib resistance in breast cancer cell lines, we first developed a signature (RBsig) from the The Cancer Genome Atlas (TCGA) dataset [3]. One of the main functions of Rb on cell cycle is repression of E2F1- and E2F2- mediated transcription; therefore the RBsig was developed by analyzing genes that correlated with E2F1 and E2F2 expression. The signature included a final set of 87 genes (Table 1) and the functional analysis, not surprisingly, revealed that the gene ontology (GO) annotations most significantly associated with the signature were cell cycle, M phase, DNA replication and nuclear division (Figure 1).

**Table 1:** Genes included in the RBsig

RBsig functional signature		
CDC45	RAD51	PIF1
TPX2	CCNB2	NDC80
CDCA5	CENPO	ASPM
CCNA2	CDT1	DEPDC1
MYBL2	DEPDC1B	NEIL3
UBE2C	NCAPH	SGOL1
CDCA3	BUB1B	KIFC1
MCM10	CENPE	MCM7
FOXM1	KIF4B	MKI67
BIRC5	PKMYT1	TTK
CENPA	DLGAP5	KIF14
AURKB	MELK	OIP5
KIF2C	KIF20A	NUSAP1
CDCA8	PTTG1	ASF1B
TICRR	TRIP13	FAM64A
ORC1	GTSE1	MND1
PLK1	PTTG3P	STIL
EXO1	SPC25	RRM2
RAD54L	CDC25A	PRC1
CEP55	CENPM	ANLN
CDC20	PTTG2	FANCI
CENPI	FAM83D	SKA1
AURKA	CENPN	SKA3
TROAP	ORC6	MCM4
POLQ	CHEK1	ARHGAP11A
KIF4A	CDKN3	KIF15
CLSPN	KIF11	AUNIP
BUB1	MTFR2	CENPW
BLM	KIF23	NUF2

**Figure 2:**
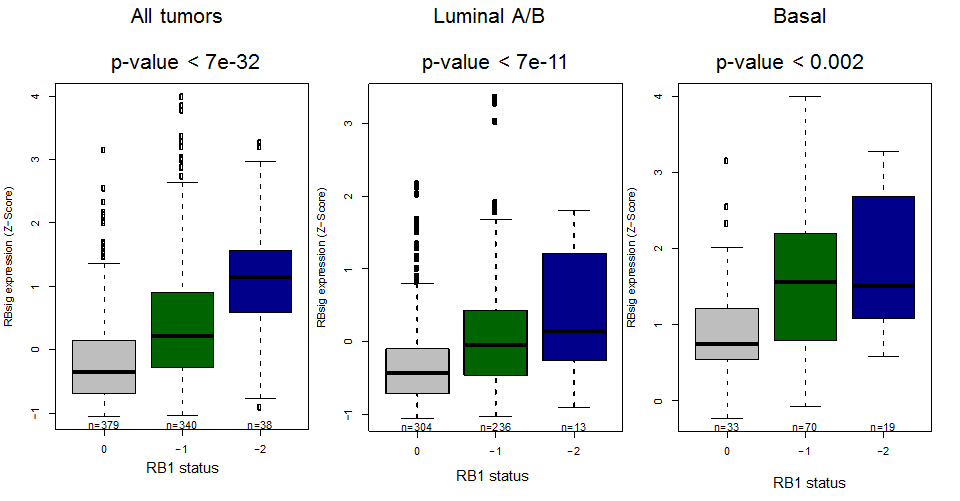
Association between RBsig expression levels and genetic RB1 status Boxplots represent the RBsig signature expression according to putative RB1 status (0= diploid, -1=heterozygous loss, -2=complete loss) in all tumors, regardless the molecular subtype and in luminal and basal subtypes in the TCGA dataset [3].

Being derived from E2F1 and E2F2 associated genes, as a first step we analyzed whether RBsig would be differentially expressed in tumors with RB1 putative diploid status compared to tumors with putative heterozygous or complete loss of RB1. Within the TCGA dataset, we found that RBsig expression significantly varied according to genetic status of RB1 in all tumors (*p* = < 7e-32) and in luminal or basal subtypes (*p* < 7e-11 and *p* < 0.002, respectively) (Figure 2).

Next, we assessed in the METABRIC dataset whether RBsig expression levels varied according to breast cancer molecular subtypes. To perform this analysis, we used the METABRIC because it comprises a larger number of samples compared to the TCGA dataset. As expected, and in accordance with previously published data with other Rb signatures [3, 17, 23], RBsig significantly varied by molecular subtype (*P* < 0.00001) (supplementary material - Ffigure S1) with the lowest levels of RBsig observed in the luminal A and normal-like subtypes and the highest levels in the basal-like subtype. Within luminal tumors the signature was highest in the luminal B subtype.

### Comparison of the RBsig with previously developed RB LOH and E2F signatures

Signatures that analyze the Rb pathway have been developed [18-21, 23, 24] and shown to be prognostic in patients with breast cancer and potentially predictive of response to chemotherapy and endocrine therapy [17, 22, 24]. In particular, two of these signatures have been developed starting from breast cancer tumors or cell lines [23, 24]. Herschkowitz et al. developed a signature of RB loss of heterozygosity (LOH), composed of 345 genes, by analyzing differentially expressed genes between RB1 LOH positive tumors versus tumors without RB1 LOH [23] while Miller et al. generated a signature of E2F activation composed of 61 genes from long-term estrogen deprived breast cancer cell lines [24]. Within the E2F signature, Miller et al. [24], based on cell cycle-related GO annotation, identified 37 genes associated with cell cycle regulation and 24 genes that were considered independent of the cell cycle [24].We aimed to investigate the overlap of the RBsig with these two signatures [23, 24]. We found that four genes were present in all three signatures; forty-five of the 87 genes in the RBsig were in common between RBsig and LOH signature, while only 7 genes of the RBsig were present in the E2F signature. We also compared these three signatures on a functional level by analyzing pathways and functions both in common among the three signatures or specific for each of them (supplementary material - Figure S2). Overall, 37 elements (pathways and functions) were in common among the three signatures, 245 elements were in common between the RBsig and LOH while only 40 elements were shared by RBsig and E2F; 260 elements were specific for the RBsig, 254 were specific for LOH and 483 for E2F (Supplementary Material - Figure S2). The list of pathways and functions in common or specific for each signature is provided in Table S2. Finally, within the METABRIC dataset, we calculated the correlation between the distributions of the expression values of the RBsig, the LOH and the E2F signatures (Supplementary Material - Figure S3). A positive correlation was found between the RBsig and the LOH signatures (*r* = 0.92, *p* < 0.001), while we observed no correlation (*r* = 0.45, *p* < 0.001) between the RBsig and the E2F signatures (Supplementary Material - Figure S3).

Overall, the functional and correlation analyses show that the RBsig and the LOH signatures, despite the different approaches used to derive the two signatures [23], display a substantial overlap in terms of genes, functions, pathways and distribution while the RBsig and the E2F signatures do not. In particular, the RBsig comprises a subset of pathways represented in the LOH signature (Table S2), mostly related to proliferation and cell cycle. This is not surprising, considering that the RBsig was developed to specifically identify genes involved in cell cycle control, which are known to be modulated by Rb, while the LOH identifies a larger number of genes induced by RB loss, including those involved in functions different from cell cycle control. On the other hand, the diversity between the RBsig and the E2F signatures might be explained by the peculiar approach used to derive the E2F signature. This was derived from analysis of two long term estrogen deprived (LTED) breast cancer cell lines [24] and is more likely related to estrogen-independent estrogen receptor (ER) activity in LTED cells rather than to the activation status of the Rb pathway.

### Prognostic role of RBsig in breast cancer patients

**Figure 3:**
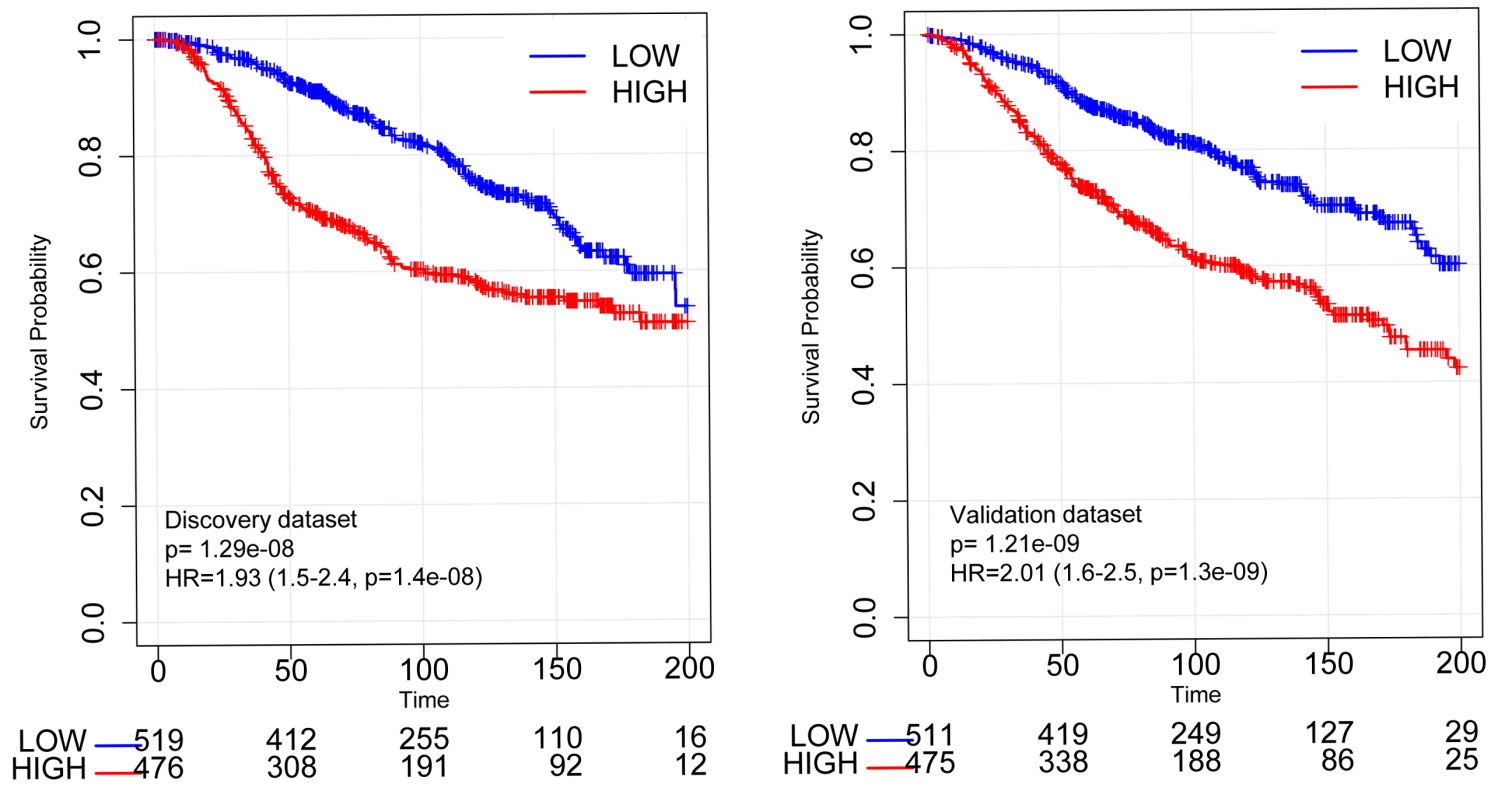
Kaplan-Meier curves according to RBsig in the METABRIC dataset Patients included in the discovery set and in the validation set of the METABRIC dataset were stratified according to RBsig expression levels using the mean as cutoff, Kaplan Meier curves were generated and HR were calculated.

To assess the prognostic value of the RBsig we conducted survival analyses on patients included in two well established gene expression datasets: the METABRIC dataset [25] and a large meta-dataset that contains clinical information such as ER status and received treatments. [26, 27]. Importantly, both datasets are independent from the one used to compute the RBsig. As expected by similar analyses with previously developed RB signatures [17, 23], the RBsig was marker of poor prognosis. In the METABRIC dataset [25], patients whose tumors were classified as having high levels of RBsig had significantly worse overall survival (OS) compared to those with low RBsig, both in the discovery (HR = 1.93, CI = 1.5-2.4, *p* = 1.4 e-08) and validation set (HR = 2.01, CI = 1.6-2.5, *p* = 1.3e-09) (Figure 3). In addition, when principal clinico-pathological characteristics such as lymph node status, tumor size, histological grade and also the LOH and E2F signatures, including cell cycle dependent and independent genes, were taken into account, the RBsig maintained an independent prognostic value (*p* = 0,0014) in a multivariate analysis (Table 2).

**Table 2:** Multivariate analysis

	HR	lower 0.95	upper 0.95	z	*p*
RBsig	2,00	1,31	3,05	3,20	0,0014
E2F Miller TW et al. [24]	0,00	0,00	68,78	−1,12	0,2645
E2F Miller TW et al. [24] CycleGenes	180,18	0,35	91482,22	1,63	0,1022
E2F Miller TW et al. [24] noCycleGenes	8,35	0,39	178,23	1,36	0,1743
LOH Herschkowitz JI et al. [23]	0,52	0,22	1,22	−1,51	0,1303
Size	1,01	1,01	1,01	6,38	1,70E-10
Grade (G3)	1,38	1,00	1,90	1,98	0,048
lymph Nodes Positive	1,06	1,05	1,07	10,35	2,00E-16

**Figure 4:**
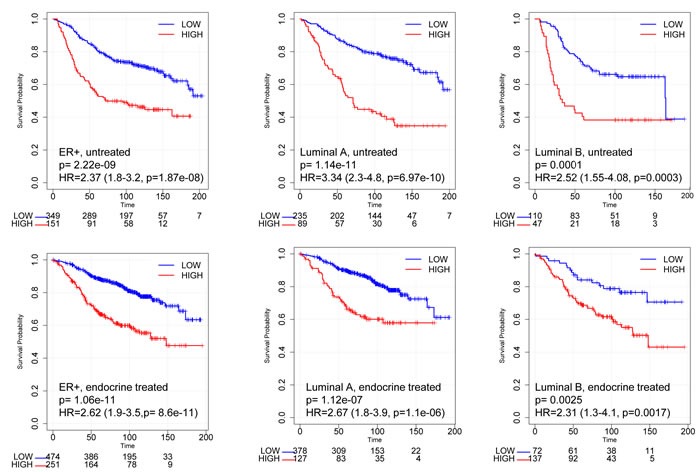
Kaplan-Meier curves according to RBsig in patients with ER+ breast cancer included in the meta-dataset Patients with ER+ tumors (untreated - upper panel; endocrine treated - bottom panel) included in the meta-dataset were stratified according to RBsig expression levels selecting the best cutoff using the best cutoff algorithm [28], Kaplan Meier curves were generated and HR were calculated.

Within the meta-dataset, using the best cut-off [28], both untreated and endocrine treated patients with ER+ tumors expressing high RBsig had significantly worse relapse-free survival (RFS) compared to those with low RBsig (HR = 2.37, CI = 1.8 − 3.2, *p* = 1.87e−08 and HR = 2.62, CI = 1.9 − 3.5, *p* = 8.6e−11, respectively) (Figure 4). Additionally, untreated or endocrine treated patients with ER+ luminal A or luminal B tumors expressing high RBsig had significantly worse RFS compared to those with low RBsig (Figure 4) (luminal A: untreated HR = 3.34, CI = 2.3 − 4.8, *p* = 6.97e−10 and endocrine treated HR = 2.67, CI = 1.8 − 3.9, *p* = 1.1e−06; luminal B: untreated HR = 2.52, CI = 1.55 − 4.08, *p* = 0.0003 and endocrine treated HR = 2.31, CI = 1.3 − 4.1, *p* = 0.0017) indicating that RBsig can provide additional information within molecular subtypes. Similar results were obtained when the 75^th^ percentile was used as cut-off for defining high versus low RBsig expression (Supplementary Material - Figure S4).

### Prediction of palbociclib resistance in breast cancer cell lines

**Figure 5:**
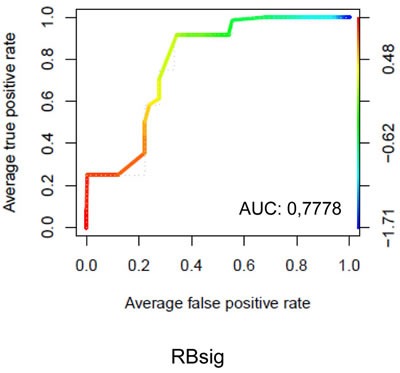
ROC curves of RBsig on all breast cancer cell lines ROC curve analysis was performed on data obtained from breast cancer cell lines analyzed by RNAseq technology. Cells were classified as sensitive or resistant to palbociclib based on the IC 50 value obtained by Finn et al [12] using 300 nanomolar (nM) as threshold

The ability of the RBsig to discriminate palbociclib resistant versus sensitive breast cancer cell lines was analyzed in a large dataset of human cancer cell lines (EGAS00001000610) [29] by taking into account IC50 data from Finn et al [12]. Cell lines with IC50 of 300 nM or more were considered resistant to palbociclib. The list of the cell lines included in the analysis with corresponding IC50 values is shown in Table S1. A relevant performance of the RBsig in identifying palbociclib resistant cell lines was obtained in this dataset with a ROC area under curve (AUC) of 0,7778 (Figure 5). It has been demonstrated that basal-like breast cancer cell lines are more frequently resistant to palbociclib [12]. Since RBsig correlates well with molecular subtypes, we hypothesized that levels of RBsig simply reflected the molecular subtype, explaining the observed prediction of palbociclib resistance. For this reason, we performed ROC curve analysis restricted to luminal breast cancer cell lines only and found that the RBsig maintained its predictive value (ROC AUC = 0,7778 Supplementary Material - Figure S5). However, this analysis was affected by the fact that, as expected based on the demonstrated sensitivity of luminal cell lines to palbociclib [12], only two luminal cell lines in the RNAseq dataset had an IC50 ≥ 300 nM. Finally, we performed ROC curve analyses for the LOH and E2F signatures. As expected based on the previous comparisons with RBsig, we found a similar performance of the LOH signature, with a AUC = 0,8194 (Supplementary Material - Figure S6), while E2F signature failed to accurately discriminate resistant versus sensitive breast cancer cell lines (ROC AUC 0,5046, data not shown).

Currently, endocrine therapy represents the mainstay of the treatment of patients with ER+/HER2neg advanced breast cancer. Endocrine agents, including aromatase inhibitors, tamoxifen and fulvestrant, are often administered as single agents and demonstrate durable responses with limited side effects [30]. Data from recent clinical trials have shown that combinations of endocrine agents and palbociclib increase the effectiveness of treatments [7-9]. However, there is lack of predictive tools to rationally allocate patients to the most active treatment.

To date, no single biomarker, other than ER+/HER2neg status, has been validated to select patients for palbociclib treatment. The lack of biomarkers of *de novo* resistance to CDK4/6 inhibitors will likely generate a scenario in the near future where most of the patients with ER+/HER2neg metastatic breast cancer, either treatment-naïve or pre-treated with endocrine therapy, will be offered a CDK4/6 inhibitor. However, data from the PALOMA-1 and the PALOMA-3 indicate that a proportion of patients do not achieve a clinical benefit (i.e. response or prolonged stabilization) from palbociclib [7-9]. A sensitive and specific biomarker of *de novo* resistance to CDK4/6 inhibitors would dramatically improve patient selection. Preclinical studies have suggested determinants of palbociclib sensitivity, including high levels of Rb and cyclinD1 (CCND1) or low levels of p16 (CDKN2A)[12, 13]. However, in PALOMA-1, patients who were included after selecting for CCND1 amplification and/or loss of p16 did not seem to show increased activity of palbociclib compared to the unselected patients for whom no molecular screening was required [8]. Additionally, in a recent Phase II trial [16] single agent palbociclib gave clinical benefit in only 21% of patients with Rb positive ER+ metastatic breast cancer, although this trial was conducted in a heavily pre-treated population. These data suggest that CCND1 amplification, p16 or Rb status alone are not sufficient for the identification of patients who are sensitive or resistant to palbociclib. Indeed, a wider analysis of the Rb-E2F pathway in breast cancer cells suggested that resistance to palbociclib might be mediated by other components of the Rb pathway [14]. Moreover, it has been demonstrated that, after palbociclib treatment, there is an attenuation of gene products associated with a signature of Rb loss, suggesting that modulation of the Rb pathway might be implicated in palbociclib response [17]. Here, for the first time, we demonstrated that RBsig is a good predictor in discriminating palbociclib sensitive vs resistant breast cancer cell lines, indicating that a wide analysis of the Rb pathway could help to identify patients who are resistant to CDK 4/6 inhibitors.

The RBsig and the LOH signatures share many similarities and a comparable performance in discriminating resistant versus sensitive breast cancer cell lines. However one advantage of using the RBsig as a predictive tool for CDK4/6 inhibitors might be the smaller number of genes comprised in this signature compared to the LOH (87 versus 345, respectively). In addition, RBsig showed a prognostic value independently from LOH signature and other clinical variables. Finally, our analyses revealed that the RBsig comprises a subset of genes and functions of the LOH. It could be hypothesized that these shared genes and functions are those most involved in palbociclib resistance.

We acknowledge that the principal limitation of our study is the lack of validation in patients receiving palbociclib or other CDK4/6 inhibitors. However to date, there are no publicly available datasets of patients treated with CDK4/6 inhibitors where we could further test this hypothesis.

In this study we demonstrated that a newly developed functional signature of Rb loss, the RBsig is able to identify breast cancer cell lines resistant to palbociclib. If the results of our study will be confirmed in breast cancer patients receiving CDK4/6 inhibitors, signatures of Rb loss-of-function might become a useful tool in identifying patients who are not going to respond to palbociclib treatment. Additional validation of gene signatures of Rb loss-of-function on cohorts of breast cancer patients treated with CDK4/6 inhibitors is warranted in the next future.

## MATERIALS AND METHODS

### Construction of the RBsig

Gene expression data from breast cancer samples in TCGA [3] were used for the construction of the RBsig. As a first step, within the TCGA breast cancer dataset, we calculated the Pearson correlations among the expression of the E2F1 or E2F2 genes and all other genes. Then we filtered out the genes with low correlation (p < 0.65) and combined the gene lists obtaining the candidate RBsig.

### Functional and correlation analyses

Functional analysis of the RBsig was performed using DAVID/EASE web tool [31, 32] with default parameters and procedures. We then filtered out the biological themes with p value less than 0.005. Enriched GO terms were plotted in a bi-dimensional space using a clustering algorithm that relies on semantic similarity measures using REVIGO tool [33].

To evaluate the association of the RBsig with the RB1 genomic status of breast cancers within the TCGA dataset, we fit a regression linear model linking the RB1 genomic status (putative diploid (0), heterozygous loss (−1), complete loss (−2) as derived by the copy-number analysis algorithm GISTIC [35] and the RBsig expression. Similarly, we measured the levels of expression of the RBsig within the different breast cancer molecular subtypes in the METABRIC dataset. Boxplots were used to represent the RBsig expression levels in each molecular subtype and the one-way analysis of variance (ANOVA) was used to determine whether there were any significant differences among them.

We compared the RBsig with two previously developed signatures that analyze the Rb pathway namely, the RB LOH signature by Herschkowitz et al. [23] and the E2F signature by Miller et al. [24]. In order to compare the different signatures from a functional point of view, we performed functional and pathway enrichment analysis using Ingenuity Pathway Analysis tool (IPA). For each signature we selected the most enriched functions and pathways (−log [pValue] > = 2, right-tailed Fisher exact test). Overlaps of functions and pathways were shown using a Venn diagram.

To examine the relationship between the different signatures (RBsig, RB LOH and E2F signatures), within the METABRIC dataset, we generated scatterplots representing the values of the two variables of interest (expression values of two different signatures) and we calculated the correlation between the distributions of signatures expression values. Gene lists were retrieved by the corresponding publications [23, 24].

All the statistical analyses were performed in R/Bioconductor environment (Version 3.1).

### Survival analysis

The prognostic value of the RBsig was analyzed in the METABRIC dataset [25]. In addition, in order to evaluate the prognostic role of the RBsig in patients with ER positive breast cancer, data deriving from a previously described meta-dataset composed of nearly 4,000 breast cancer samples [26, 27, 34] were analyzed. This meta-dataset includes gene expression data obtained by Affymetrix microarray technology [26, 27, 34]. Samples were classified according to the expression of the RBsig as low or high, selecting the best cut-off [28]. Clinical information such as ER status, molecular subtype and systemic treatments were used to stratify the different cohorts of patients in order to examine correlations between the signatures, molecular subtypes (Luminal A, Luminal B, Basal and HER2 enriched) and RFS.

### Multivariate analysis

Multivariate analysis was performed on the above-mentioned cohort of patients within the METABRIC dataset [25] using the Cox proportional hazards regression modelling. In this analysis we searched for the relationships between the RBsig and other clinically significant variables (size, grade and lymph-node status). To specifically compare RBsig to the other associated gene signatures (LOH and E2F), we included them in the multivariate analysis.

### Prediction of the signatures and ROC curve analysis

In order to analyze the ability of the RBsig, LOH and E2F signatures to discriminate resistant versus sensitive breast cancer cell lines, we used data from breast cancer cell lines analyzed by RNAseq technology derived from 675 human cancer cell lines (EGAS00001000610) [29]. From this dataset we extracted data relating to all breast cancer cell lines as well as to luminal-only breast cancer cell lines. Cells were classified as sensitive or resistant to palbociclib based on the IC 50 value obtained by Finn et al [12] using 300 nanomolar (nM) as threshold. ROC curves were generated using the signatures as two different classifiers using the METABRIC dataset [25]. Expression data were downloaded and normalized using R/Bioconductor environment (Version 3.1); ROC analyses were performed using pROC and ROCR packages.

## SUPPLEMENTARY MATERIALS FIGURES AND TABLES







## Abbreviations

HR: hormone receptor
HER2: human epidermal growth factor receptor type 2
Rb: Retinoblastoma gene product
LOH: loss of heterozygosity
CDK4/6: Cyclin Dependent Kinases 4 and 6
FDA: Food and Drug Administration
TCGA: The Cancer Genome Atlas
IPA: Ingenuity Pathway Analysis
ER: estrogen receptor
RFS: relapse-free survival
OS: overall survival
HR: Hazard Ratio
nM: nanomolar
GO: gene ontology
LTED: long term estrogen deprived
AUC: area under curve
