# Supplementary material for: A gene expression signature of retinoblastoma loss-of-function is a predictive biomarker of resistance to palbociclib in breast cancer cell lines and is prognostic in patients with ER positive early breast cancer
Source: Oncotarget. 2016 Sep 13;7(42):68012–22. doi: 10.18632/oncotarget.12010 (PMC5356535; doi:10.18632/oncotarget.12010)
Supplement: Supplementary file 1 [file oncotarget-07-68012-s001.pdf]

A gene expression signature of Retinoblastoma loss-of-function is a predictive biomarker of resistance to palbociclib in breast cancer cell lines and is prognostic in patients with ER positive early breast cancer

Supplementary Material

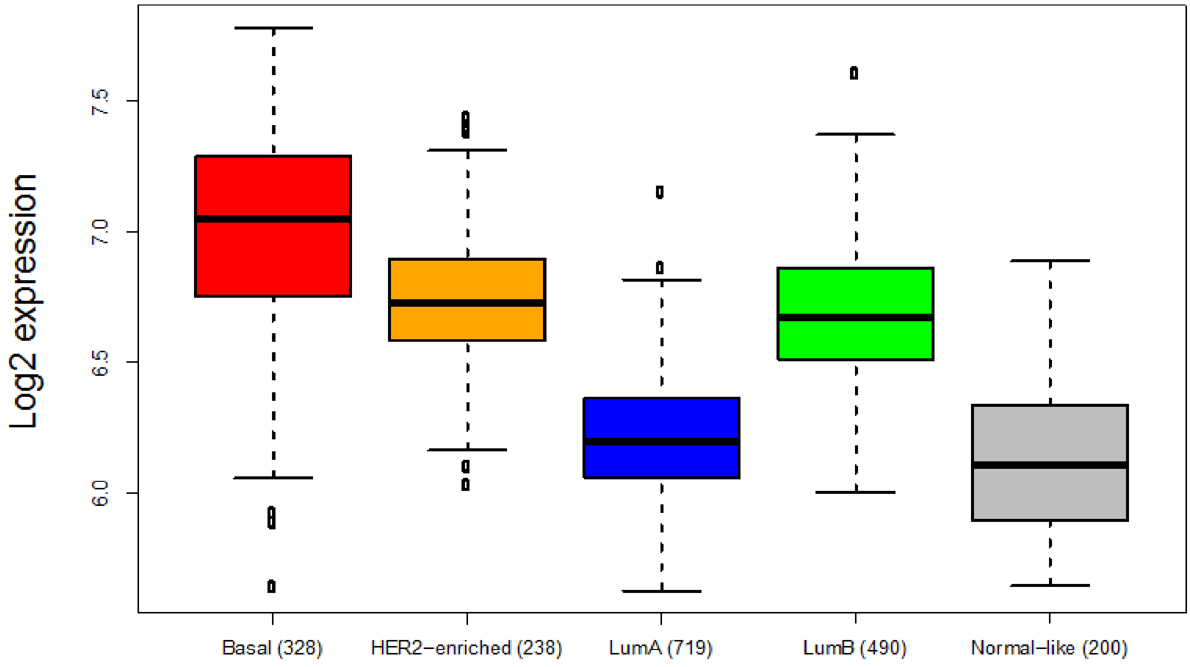

Figure S1: **RBsig and molecular subtypes:** Boxplots represent the RBsig signature expression in each breast cancer subtype within the METABRIC dataset.

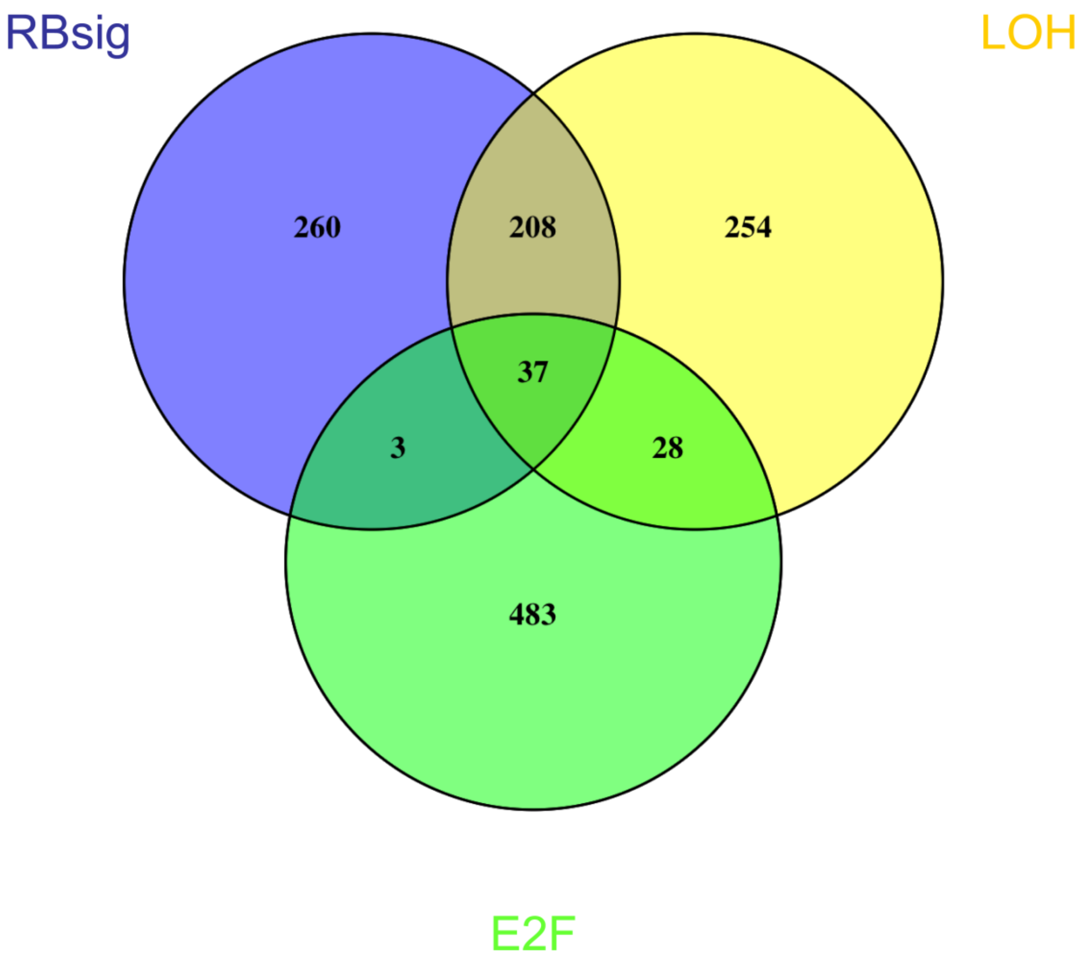

Figure S2: Pathways and Functional comparison of the RBsig, LOH and E2F signatures: Overlaps of the most enriched pathways and functions in the RBsig, LOH and E2F signatures are shown in the Venn diagram.

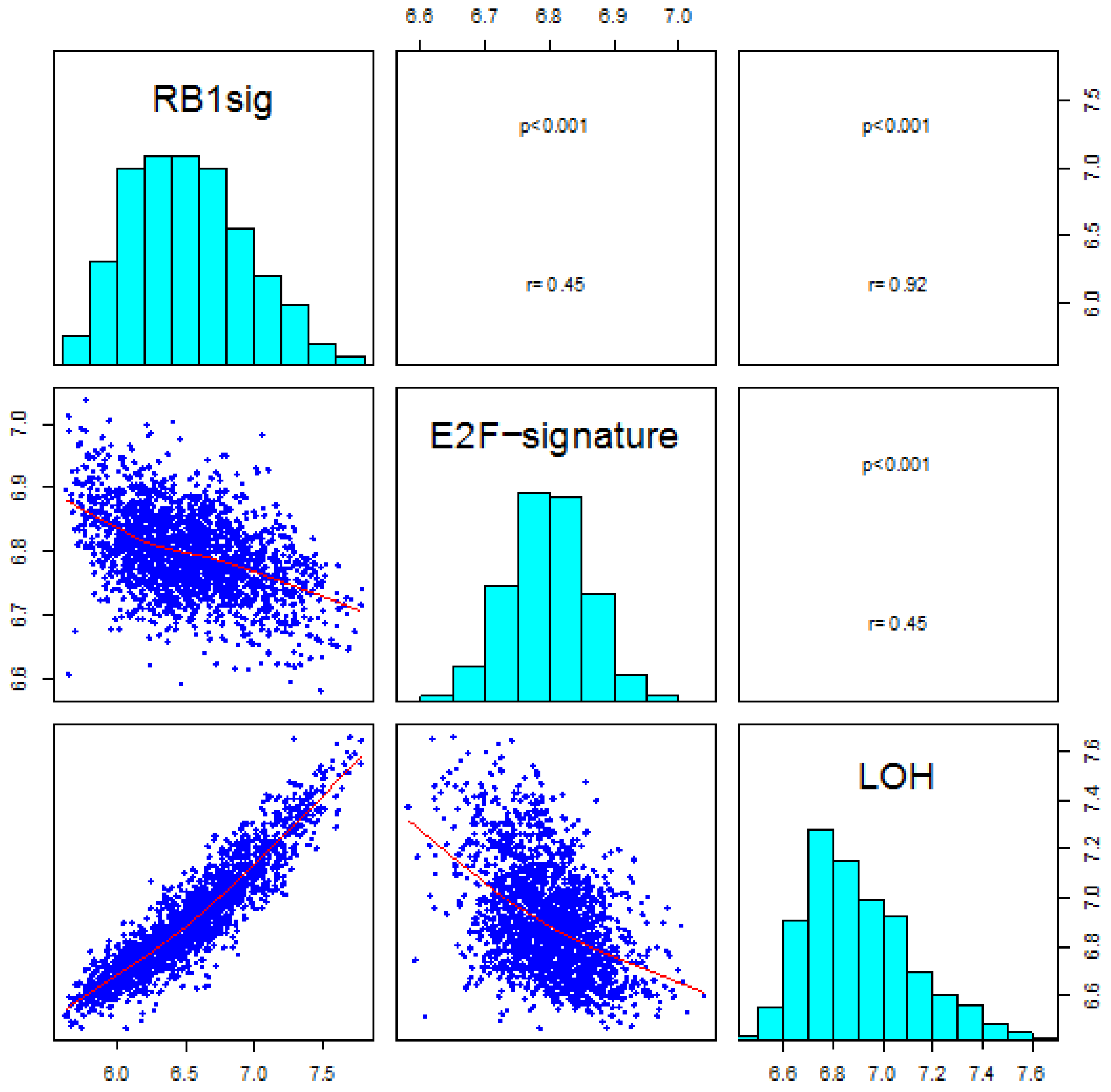

Figure S3: Correlation between RBsig, LOH [23] and E2F [24] signatures: in the METABRIC dataset scatterplots representing the values of the two variables of interest (expression values of two different signatures) were generated and the correlation between the distributions of signatures expression values was calculated.

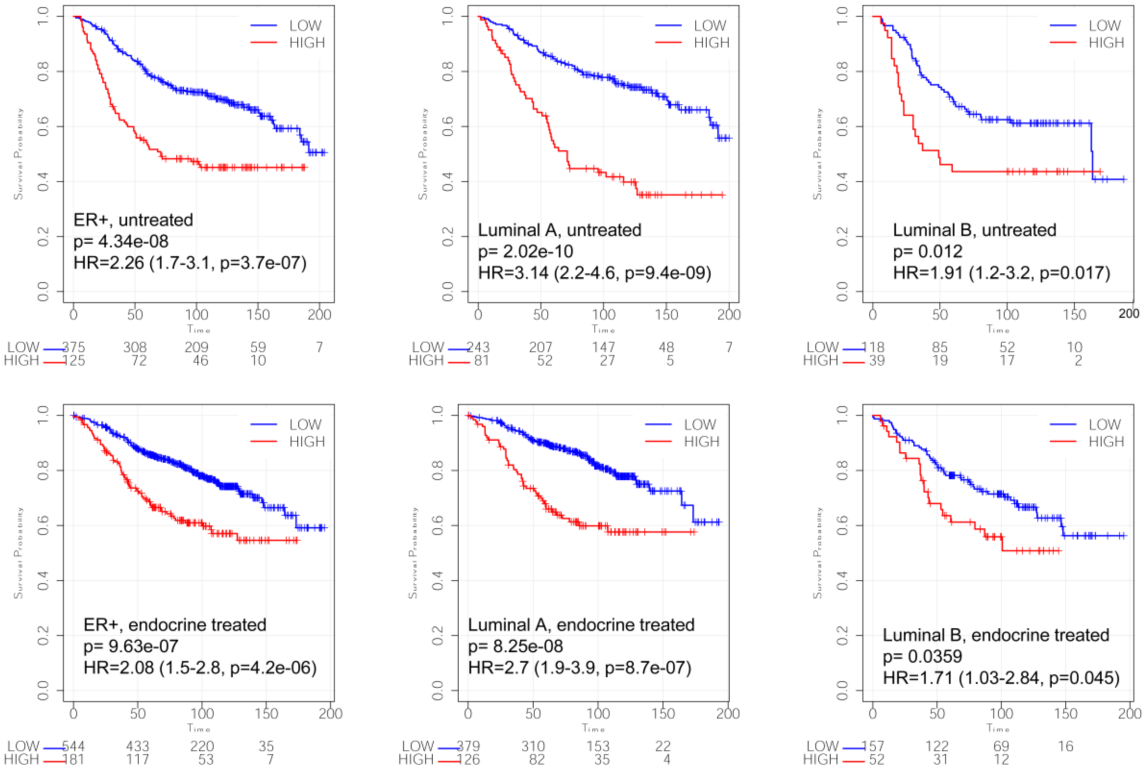

Figure S4: Kaplan-Meier curves according to RBsig using the 75th percentile as cut-off in patients with ER+ breast cancer included in the meta-dataset Patients with ER+ tumors (untreated - upper panel; endocrine treated - lower panel) included in the metadataset were stratified according to RBsig expression levels using the 75th percentile, Kaplan Meier curves were generated and HR were calculated.

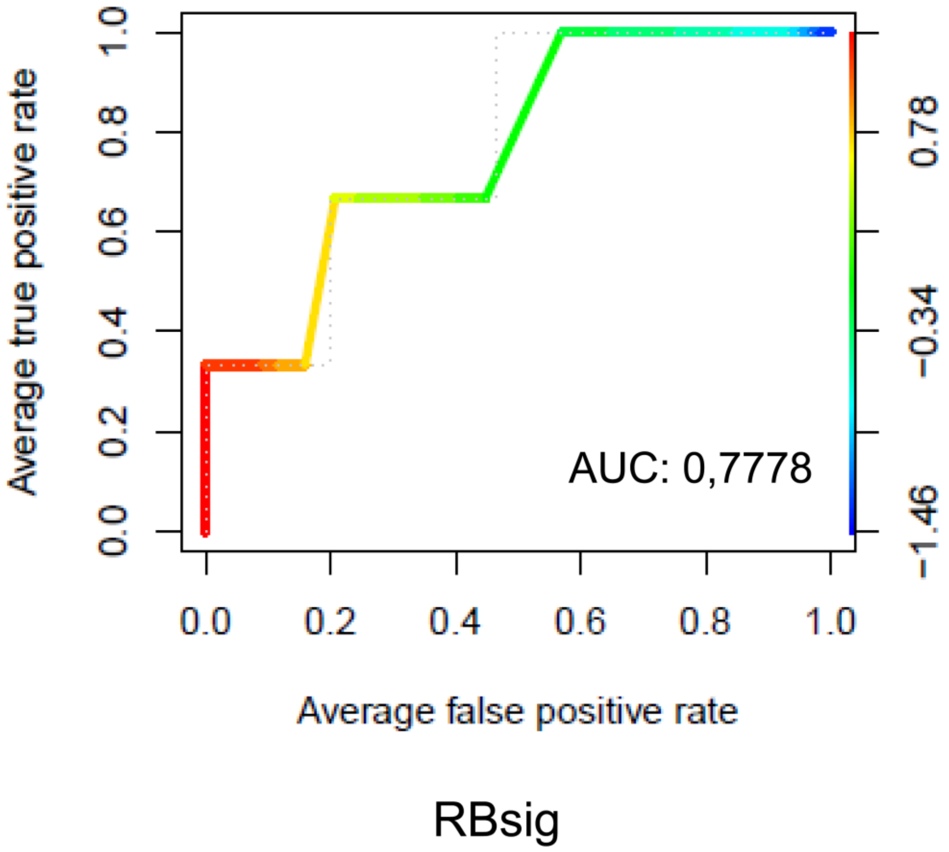

Figure S5: ROC curves of RBsig signature on luminal breast cancer cell lines: ROC curve analysis was performed on data obtained from luminal breast cancer cell lines analyzed by RNAseq technology. Cells were classified as sensitive or resistant to palbociclib based on the IC 50 value obtained by Finn et al [12] using 300 nanomolar (nM) as threshold.

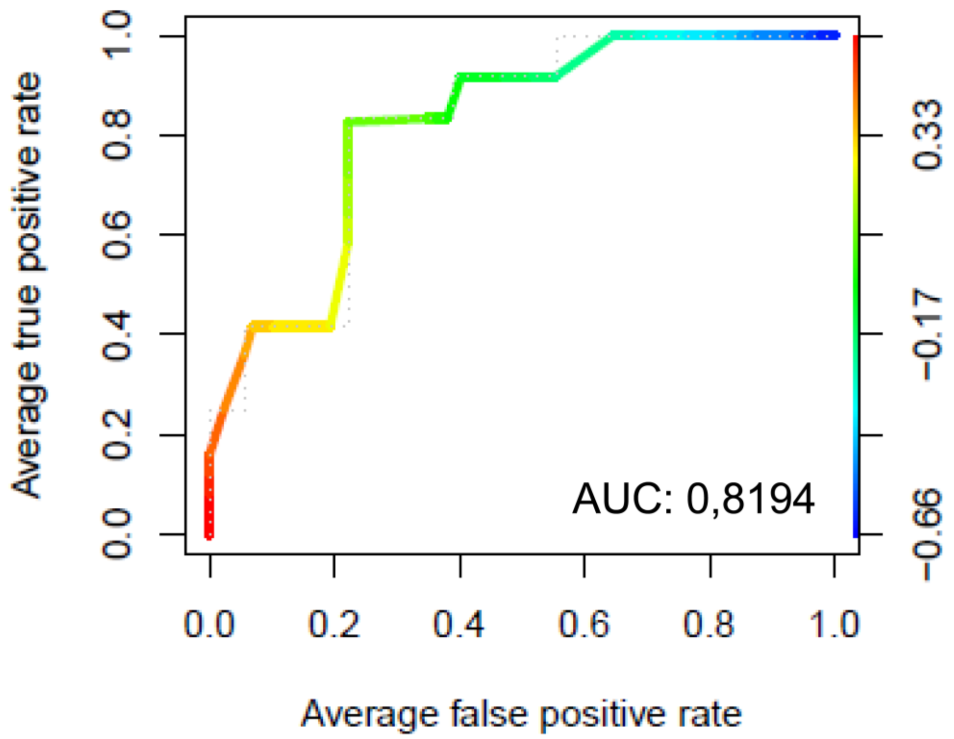

LOH

Figure S6: ROC curves of LOH signature [23] on all breast cancer cell lines ROC curve analysis was performed on data obtained from breast cancer cell lines analyzed by RNAseq technology. Cells were classified as sensitive or resistant to palbociclib based on the IC 50 value obtained by Finn et al [12] using 300 nanomolar (nM) as threshold.
