# Supplementary material for: A gene expression signature of retinoblastoma loss-of-function is a predictive biomarker of resistance to palbociclib in breast cancer cell lines and is prognostic in patients with ER positive early breast cancer
Source: Oncotarget. 2016 Sep 13;7(42):68012–22. doi: 10.18632/oncotarget.12010 (PMC5356535; doi:10.18632/oncotarget.12010)
Supplement: Supplementary file 2 [file oncotarget-07-68012-s002.docx]

| **Cell line** | **type** | **IC50** | **Sensitivity** |
| --- | --- | --- | --- |
| MDA-MB-175 | Luminal | 4 | sensitive |
| ZR-75-30 | Luminal | 5 | sensitive |
| CAMA-1 | Luminal | 8 | sensitive |
| MDA-MB-134 | Luminal | 13 | sensitive |
| HCC-202 | Luminal | 21 | sensitive |
| UACC-893 | Luminal | 24 | sensitive |
| MDA-MB-361 | Luminal | 44 | sensitive |
| HCC-1500 | Luminal | 45 | sensitive |
| HCC-1419 | Luminal | 51 | sensitive |
| HCC-38 | Basal | 64 | sensitive |
| MDA-MB-415 | Luminal | 64 | sensitive |
| MCF-10A | Luminal | 92 | sensitive |
| UACC-812 | Luminal | 96 | sensitive |
| HCC-2218 | Luminal | 100 | sensitive |
| ZR-75-1 | Luminal | 110 | sensitive |
| MDA-MB-453 | Luminal | 115 | sensitive |
| MCF-7 | Luminal | 148 | sensitive |
| BT-20 | Basal | 177 | sensitive |
| SK-BR-3 | Luminal | 300 | resistant |
| KPL-1 | Luminal | 327 | resistant |
| HCC-1143 | Basal | 359 | resistant |
| HCC-1395 | Post-EMT | 472 | resistant |
| BT-549 | Post-EMT | 1000 | resistant |
| DU4475 | Basal | 1000 | resistant |
| HCC-1187 | Basal | 1000 | resistant |
| HCC-1954 | Basal | 1000 | resistant |
| HCC-70 | Basal | 1000 | resistant |
| MDA-MB-157 | Post-EMT | 1000 | resistant |
| MDA-MB-436 | Post-EMT | 1000 | resistant |
| MDA-MB-468 | Basal | 1000 | resistant |
