# Supplementary material for: A gene expression signature of retinoblastoma loss-of-function is a predictive biomarker of resistance to palbociclib in breast cancer cell lines and is prognostic in patients with ER positive early breast cancer
Source: Oncotarget. 2016 Sep 13;7(42):68012–22. doi: 10.18632/oncotarget.12010 (PMC5356535; doi:10.18632/oncotarget.12010)
Supplement: Supplementary file 3 [file oncotarget-07-68012-s003.docx]

**Functions overlap**

| **36 common elements in "RB1", "LOH" and "E2F":** | | **201 common elements in "RB1" and "LOH":** | **27 common elements in "LOH" and "E2F":** | **3 common elements in "RB1" and "E2F":** | **260 elements included exclusively in "RB1":** | **236 elements included exclusively in "LOH":** | **434 elements included exclusively in "E2F":** |
| --- | --- | --- | --- | --- | --- | --- | --- |
| cell cycle progression | | mitosis | transcription of RNA | proliferation of tumor cells | spindle checkpoint of tumor cell lines | delay in mitosis of tumor cell lines | transactivation of RNA |
| benign neoplasia | | mitosis of tumor cell lines | proliferation of connective tissue cells | growth of malignant tumor | chromosomal congression of tumor cell lines | DNA recombination | activation of DNA endogenous promoter |
| proliferation of cells | | arrest in mitosis | development of central nervous system | breast adenocarcinoma | mitotic exit | primary carcinoma | transcription of DNA |
| cell proliferation of tumor cell lines | | segregation of chromosomes | growth of connective tissue |  | cohesion of sister chromatids | binding of chromatin | binding of DNA |
| apoptosis of tumor cell lines | | mitosis of cervical cancer cell lines | abnormal morphology of embryonic tissue |  | chromosomal congression of cervical cancer cell lines | morbidity or mortality | binding of protein binding site |
| necrosis | | M phase | cell death of connective tissue cells |  | S phase of cervical cancer cell lines | cell death of breast cancer cell lines | uterine leiomyoma |
| apoptosis | | arrest in mitosis of tumor cell lines | hyperplasia of secretory structure |  | quantity of chromosomes | migration of neurons | differentiation of connective tissue |
| interphase | | uterine serous papillary cancer | hyperplasia of tissue |  | pelvic tumor | abdominal neoplasm | smooth muscle tumor |
| cell death | | female genital tract serous cancer | cell proliferation of breast cancer cell lines |  | abnormal morphology of mitotic spindle | arrest in interphase of fibroblast cell lines | development of body trunk |
| organismal death | | M phase of tumor cell lines | arrest in proliferation of cells |  | arrest in metaphase | apoptosis of thymocytes | morphology of body cavity |
| cell survival | | arrest in mitosis of cervical cancer cell lines | invasion of tumor cell lines |  | unwinding of DNA | hyperplasia of genital organ | differentiation of cells |
| cell viability | | cell viability of tumor cell lines | apoptosis of breast cancer cell lines |  | elongation of mitotic spindle | digestive organ tumor | leiomyomatosis |
| arrest in interphase | | serous neoplasm | invasion of cells |  | arrest in M phase of cervical cancer cell lines | abnormal morphology of blastocyst | benign neoplasm of female genital organ |
| repair of DNA | | M phase of cervical cancer cell lines | size of embryo |  | liver cancer | abnormal morphology of cerebral cortex | benign connective or soft tissue neoplasm |
| mammary tumor | | organization of mitotic spindle | Growth Failure |  | hepatocellular carcinoma | incidence of lymphoma | abnormal morphology of body cavity |
| arrest in cell cycle progression | | cell death of tumor cell lines | carcinoma in lung |  | entry into S phase of cervical cancer cell lines | neoplasia of epithelial tissue | synthesis of steroid |
| interphase of tumor cell lines | | alignment of chromosomes | cell death of fibroblast cell lines |  | separation of sister chromatids | meiosis I of germ cells | development of genitourinary system |
| breast or ovarian cancer | | checkpoint control | abnormal morphology of extraembryonic tissue |  | organization of chromosomes | benign ovarian tumor | synthesis of lipid |
| female genital neoplasm | | delay in mitosis | cell death of tumor cells |  | urinary tract cancer | colon tumor | development of abdomen |
| tumorigenesis of reproductive tract | | cell death of cervical cancer cell lines | growth of organism |  | mitosis of epithelial cell lines | tumorigenesis of malignant tumor | growth of epithelial tissue |
| genital tumor | | G2 phase | cell death of cancer cells |  | proliferation of embryonic tissue | colorectal neoplasia | size of cells |
| tumorigenesis of genital organ | | formation of mitotic spindle | incidence of tumor |  | condensation of mitotic chromosomes | fusion of chromosomes | formation of brain |
| adenoma | | ploidy | apoptosis of tumor cells |  | formation of midzone | prometaphase of tumor cell lines | quantity of steroid |
| growth of tumor | | quantity of mitotic spindle | vascular tumor |  | missegregation of sister chromatids | primary ovarian cancer | quantity of cells |
| connective or soft tissue tumor | | apoptosis of cervical cancer cell lines | cell proliferation of fibroblasts |  | mitotic exit DNA damage checkpoint of cells | apoptosis of colorectal cancer cell lines | differentiation of red blood cells |
| breast cancer | | delay in mitosis of cervical cancer cell lines | migration of tumor cell lines |  | G2 phase of cervical cancer cell lines | thymus gland tumor | differentiation of epithelial cells |
| uterine tumor | | cytokinesis | respiratory system tumor |  | arrest in G1/S phase transition of tumor cell lines | thoracic neoplasm | development of head |
| arrest in interphase of tumor cell lines | | arrest in M phase |  |  | entry into S phase of tumor cell lines | serous ovarian carcinoma | migration of cells |
| cell cycle progression of tumor cell lines | | S phase |  |  | G2 phase of bone cancer cell lines | Gastrointestinal Tract Cancer and Tumors | cell movement |
| proliferation of lung cancer cell lines | | prometaphase |  |  | formation of chromosome components | interphase of fibroblast cell lines | cell death of blood cells |
| cell proliferation of carcinoma cell lines | | G2/M phase |  |  | cell viability of myeloma cell lines | colony survival of cells | development of reproductive system |
| lung tumor | | chromosomal congression of chromosomes |  |  | cohesion of centromeres | large intestine neoplasm | steroid metabolism |
| G1 phase | | morphology of mitotic spindle |  |  | delay in initiation of mitotic entry of cervical cancer cell lines | recombination of cells | estrous cycle |
| cell death of colorectal cancer cell lines | | spindle checkpoint of cells |  |  | premature chromatid separation trait | ploidy of epithelial cells | proliferation of epithelial cells |
| hemangioblastoma | | arrest in G2 phase |  |  | tetraploidy | proliferation of lung cell lines | abnormal morphology of head |
| hemangioma | | cytokinesis of tumor cell lines |  |  | G1/S phase transition of tumor cell lines | growth of digestive organ tumor | proliferation of smooth muscle cells |
|  | | mitosis of fibroblast cell lines |  |  | recruitment of protein | thoracic cancer | differentiation of tumor cell lines |
|  | | missegregation of chromosomes |  |  | senescence of fibroblast cell lines | endometrium tumor | synthesis of terpenoid |
|  | | polyploidization |  |  | cleavage of cells | arrest in prometaphase of tumor cell lines | quantity of neurons |
|  | | ploidy of cells |  |  | abdominal adenocarcinoma | thymoma | differentiation of connective tissue cells |
|  | | attachment of spindle fibers |  |  | growth of hepatocellular carcinoma | interphase of fibroblasts | concentration of lipid |
|  | | attachment of kinetochores |  |  | mitotic index | incidence of malignant tumor | concentration of hormone |
|  | | entry into interphase |  |  | arrest in metaphase of embryonic cells | entry into mitosis | apoptosis of neurons |
|  | | female genital tract cancer |  |  | arrest in mitosis of tumor cells | alignment of sister chromatids | cellular homeostasis |
|  | | metabolism of DNA |  |  | delay in cell cycle progression of cervical cancer cell lines | formation of embryoblast | quantity of connective tissue |
|  | | cell viability of cervical cancer cell lines |  |  | formation of cleavage furrow | senescence of bone cancer cell lines | cell death of immune cells |
|  | | meiosis I of female germ cells |  |  | separation of mitotic centrosome | abnormal cell cycle | development of digestive system |
|  | | DNA replication |  |  | hematological neoplasia | tumorigenesis of lung carcinoma | uptake of lipid |
|  | | S phase of tumor cell lines |  |  | aneuploidization of fibroblast cell lines | tumorigenesis of lymphoma | quantity of blood cells |
|  | | DNA replication checkpoint |  |  | arrest in mitosis of colorectal cancer cell lines | arrest in interphase of carcinoma cell lines | differentiation of muscle cells |
|  | | cytokinesis of cervical cancer cell lines |  |  | attachment of chromosomes | intestinal adenoma | concentration of cholesterol |
|  | | G2/M phase transition |  |  | exit from M phase | growth of embryo | adipogenesis |
|  | | survival of Saccharomyces cerevisiae |  |  | fragmentation of spindle pole | serous adenocarcinoma | generation of cells |
|  | | polyploidization of cells |  |  | migration of non-small-cell lung cancer cells | initiation of interphase | inflammation of organ |
|  | | non-small cell lung cancer |  |  | organization of meiotic spindles | prometaphase of cervical cancer cell lines | morphology of cells |
|  | | entry into interphase of oocytes |  |  | segregation of homologous chromosomes | clear cell ovarian cancer | proliferation of muscle cells |
|  | | interphase of bone cancer cell lines |  |  | apoptosis of squamous cell carcinoma cell lines | quantity of benign tumor | abnormal morphology of respiratory system |
|  | | arrest in prometaphase |  |  | bladder cancer | mucinous ovarian cancer | morphology of digestive system |
|  | | uterine cancer |  |  | hematologic cancer | degeneration of embryonic tissue | differentiation of blood cells |
|  | | urogenital cancer |  |  | soft tissue sarcoma cancer | mucinous neoplasm | metastasis |
|  | | arrest in cell cycle progression of tumor cell lines |  |  | cell cycle progression of bone cancer cell lines | colorectal adenoma | necrosis of epithelial tissue |
|  | | delay in initiation of M phase |  |  | S phase checkpoint control of cervical cancer cell lines | delay in senescence of cells | atresia of ovarian follicle |
|  | | endometrial cancer |  |  | arrest in S phase of cervical cancer cell lines | initiation of mitosis | apoptosis of blood cells |
|  | | morphology of cytoskeleton |  |  | mitosis of cardiomyocytes | damage of chromosomes | invasion of breast cancer cell lines |
|  | | segregation of sister chromatids |  |  | movement of chromosomes | ovarian adenocarcinoma | development of vasculature |
|  | | cell death of melanoma cell lines |  |  | organization of asters | S phase of fibroblasts | apoptosis of connective tissue cells |
|  | | death of embryo |  |  | interphase of connective tissue cells | arrest in G1 phase of bone cancer cell lines | quantity of leukocytes |
|  | | recombination |  |  | length of microtubules | arrest in G1 phase of sarcoma cell lines | angiogenesis |
|  | | genital tract cancer |  |  | mitosis of embryonic cell lines | tumorigenesis of liver tumor | abnormal morphology of thoracic cavity |
|  | | DNA damage |  |  | replication of plasmid DNA | primary serous ovarian carcinoma | proliferation of cancer cells |
|  | | senescence of cells |  |  | excision repair | degeneration of embryoblast | quantity of adipose tissue |
|  | | quantity of centrosome |  |  | growth of carcinoma | interphase of colorectal cancer cell lines | vasculogenesis |
|  | | breast or colorectal cancer |  |  | renal clear cell adenocarcinoma | chromosomal aberration | adipogenesis of fibroblast cell lines |
|  | | digestive system cancer |  |  | assembly of kinetochores | arrest in growth of embryo | quantity of LH in blood |
|  | | abdominal cancer |  |  | accumulation of cervical cancer cell lines | atrophy of testis | abnormal morphology of digestive system |
|  | | binding of chromosome components |  |  | uterine carcinoma | cell survival of tumor cell lines | quantity of epithelial tissue |
|  | | quantity of nucleus |  |  | myosarcoma | epithelial ovarian cancer | cytostasis |
|  | | regulation of cyclin-dependent protein kinase |  |  | stabilization of microtubules | endometrioid ovarian cancer | migration of breast cancer cell lines |
|  | | interphase of cervical cancer cell lines |  |  | G1/S phase transition of cervical cancer cell lines | lymphomagenesis | morphology of connective tissue |
|  | | length of mitotic spindle |  |  | autosomal recessive primary microcephaly | tumorigenesis of carcinoma | binding of AP1 consensus site |
|  | | mitosis of colorectal cancer cell lines |  |  | density of microtubules | cell death of fibroblasts | quantity of LH |
|  | | anaphase |  |  | DNA damage checkpoint | quantity of tumor cell lines | proliferation of blood cells |
|  | | arrest in G1/S phase transition |  |  | transition of cells | tumorigenesis of hepatocellular carcinoma | cell movement of tumor cell lines |
|  | | meiosis of oocytes |  |  | microtubule dynamics | primary solid tumor | Hypertrophy |
|  | | organization of cytoplasm |  |  | bladder carcinoma | cell death of leukemia cell lines | neuronal cell death |
|  | | meiosis I of oocytes |  |  | glioblastoma cancer | exchange of sister chromatids | feeding |
|  | | G1/S phase transition |  |  | M phase of fibroblast cell lines | arrest in G2 phase of tumor cells | behavior |
|  | | arrest in S phase |  |  | adenocarcinoma in endometrium | primary endometrioid ovarian carcinoma | abnormal morphology of abdomen |
|  | | epithelial cancer |  |  | renal cell cancer | sensitivity of cells | perinatal death |
|  | | G2 phase of tumor cell lines |  |  | Renal Cancer and Tumors | cell death of embryonic stem cells | abnormal morphology of brain |
|  | | double-stranded DNA break repair |  |  | endometrioid carcinoma | arrest in G2 phase of fibroblast cell lines | anemia |
|  | | re-replication of DNA |  |  | sensitization of cells | delay in formation of tumor | quantity of lymphocytes |
|  | | gastrointestinal tract cancer |  |  | entry into mitosis of cervical cancer cell lines | homologous recombination repair of DNA | cell death of epithelial cells |
|  | | homologous recombination |  |  | mitosis of kidney cell lines | ploidy of tumor cell lines | formation of gland |
|  | | arrest in G2 phase of tumor cell lines |  |  | prophase | morphology of nucleus | differentiation of epithelial tissue |
|  | | meiosis of female germ cells |  |  | transition of thymocytes | non-M3 acute myeloid leukemia | binding of DNA fragment |
|  | | segregation of mitotic sister chromatids |  |  | lymphoid cancer | acral lentiginous melanoma cancer | development of genital organ |
|  | | arrest in G2/M phase |  |  | chronic myelomonocytic leukemia | quantity of bone cancer cell lines | differentiation of adipocytes |
|  | | mitosis of sarcoma cell lines |  |  | duplication of centrosome | quantity of sarcoma cell lines | apoptosis of epithelial cells |
|  | | Meier-Gorlin syndrome |  |  | formation of nuclear foci | Gleason's score 7 prostate cancer | inflammation of intestine |
|  | | quantity of chromosome components |  |  | multinucleation of cervical cancer cell lines | ploidy of colorectal cancer cell lines | signal transduction |
|  | | colorectal cancer |  |  | cell proliferation of kidney cell lines | initiation of S phase | proliferation of immune cells |
|  | | meiosis of germ cells |  |  | G2/M phase of cervical cancer cell lines | primary tumor | binding of thyroid hormone response element |
|  | | aneuploidy |  |  | gastrointestinal carcinoma | cell movement of neurons | apoptosis of hematopoietic cells |
|  | | microcephaly |  |  | multinucleation of cells | nasopharyngeal cancer | formation of mammary gland |
|  | | metaphase/anaphase transition |  |  | arrest in cell cycle progression of bone cancer cell lines | cell viability of lung cancer cell lines | development of leukocytes |
|  | | malignant neoplasm of large intestine |  |  | arrest in cell cycle progression of sarcoma cell lines | arrest in S phase of tumor cell lines | synthesis of reactive oxygen species |
|  | | spindle checkpoint of cervical cancer cell lines |  |  | development of lung tumor | lymphatic node tumor | proliferation of keratinocytes |
|  | | non small cell lung adenocarcinoma |  |  | quantity of peripheral T lymphocyte | cell death of myeloma cell lines | morphology of liver |
|  | | pelvic cancer |  |  | muscle tumor | cell viability of fibroblasts | quantity of interneurons |
|  | | cancer |  |  | proliferation of bone marrow cells | neoplasia of lymphoid organ | differentiation of embryonic cells |
|  | | colon cancer |  |  | activation of cytarabine | proliferation of fibroblast cell lines | metastatic solid tumor |
|  | | spindle checkpoint of mitotic cells |  |  | arrest in G2 phase of fibroblasts | osteosarcoma | growth of ovarian follicle |
|  | | progression of replication fork |  |  | formation of micronuclei | interphase of tumor cells | hypoplasia of organ |
|  | | formation of spindle pole |  |  | homologous recombination repair of tumor cell lines | aneuploidization of cells | abnormal morphology of cells |
|  | | cell cycle progression of cervical cancer cell lines |  |  | polyploidy of cells | arrest in G1 phase of fibroblasts | Edema |
|  | | entry into mitosis of tumor cell lines |  |  | premature senescence of fibroblasts | cell survival of lung cancer cell lines | transdifferentiation of cells |
|  | | ovarian cancer |  |  | maintenance of telomeres | metaphase | abnormal morphology of nervous system |
|  | | cell death of sarcoma cell lines |  |  | arrest in interphase of epithelial cells | cell proliferation of colorectal cancer cell lines | length of ventricular zone |
|  | | malignant solid tumor |  |  | separation of postcytokinetic cells | hereditary neoplastic syndrome | inflammation of body region |
|  | | formation of microtubules |  |  | myeloid neoplasm | Hereditary Nonpolyposis Colorectal Cancer | proliferation of intestinal cells |
|  | | organization of cytoskeleton |  |  | cell viability of prostate cancer cell lines | accumulation of colorectal cancer cell lines | quantity of epithelial cells |
|  | | S phase of bone cancer cell lines |  |  | cell proliferation of brain cancer cell lines | delay in anaphase of tumor cell lines | fertility |
|  | | prostatic intraepithelial neoplasia |  |  | processing of replication fork | nodular melanoma | transcription of mRNA |
|  | accumulation of cells |  |  | malignant neoplasm of endocrine gland | primary clear cell ovarian carcinoma | cardiogenesis |  |
|  | cell viability of breast cancer cell lines |  |  | migratory capacity of tumor cell lines | senescence of sarcoma cell lines | synthesis of progesterone |  |
|  | entry into S phase |  |  | bone marrow cancer | somatic hypermutation | gonadogenesis |  |
|  | arrest in G1 phase |  |  | cohesion of mitotic sister chromatids | superficial spreading melanoma | degeneration of nervous system |  |
|  | aneuploidy of cells |  |  | proliferation of embryoblast | S phase of fibroblast cell lines | Bleeding |  |
|  | condensation of chromosomes |  |  | central nervous system cancer | cell viability of carcinoma cell lines | T cell development |  |
|  | arrest in prometaphase of cervical cancer cell lines |  |  | senescence of breast cancer cell lines | invasion of tumor | colitis |  |
|  | accumulation of tumor cell lines |  |  | premature senescence of fibroblast cell lines | re-entry into cell cycle progression | size of connective tissue |  |
|  | delay in initiation of anaphase |  |  | endometriosis | arrest in G2 phase of endometrial cancer cell lines | developmental delay |  |
|  | arrest in interphase of fibroblasts |  |  | gastrointestinal adenocarcinoma | arrest in mitosis of breast cancer cell lines | mass of gonad |  |
|  | cycling of centrosome |  |  | glandular intraepithelial neoplasm | arrest in mitosis of fibrosarcoma cell lines | concentration of triacylglycerol |  |
|  | senescence of fibroblasts |  |  | pituitary gland adenoma | arrest in spindle checkpoint of cells | loss of hair |  |
|  | association of chromosome components |  |  | embryonal tumor | blebbing of nucleus | synthesis of fatty acid |  |
|  | leiomyosarcoma |  |  | Bloom's syndrome | delay in initiation of mitosis of sarcoma cell lines | recognition memory |  |
|  | abnormal morphology of embryoblast |  |  | DNA replication checkpoint of cervical cancer cell lines | delay in mitosis of bone cancer cell lines | degeneration of neurons |  |
|  | arrest in interphase of bone cancer cell lines |  |  | DNA replication checkpoint of colorectal cancer cell lines | entry into S phase of epidermal cells | colony formation of cells |  |
|  | sarcoma |  |  | DNA replication checkpoint of fibroblast cell lines | function of fibroblast-like synoviocytes | differentiation of cardiomyocytes |  |
|  | arrest in G2 phase of bone cancer cell lines |  |  | G2 phase of lymphoblastoid cell lines | patterning of neuronal progenitor cells | secretion of molecule |  |
|  | entry into mitosis of colorectal cancer cell lines |  |  | Meier-Gorlin syndrome type 1 | replication of leukemia cell lines | development of exocrine gland |  |
|  | amplification of centrosome |  |  | Meier-Gorlin syndrome type 3 | checkpoint control of tumor cell lines | folliculogenesis |  |
|  | adenocarcinoma |  |  | Meier-Gorlin syndrome type 4 | ploidy of lymphatic system cells | Neurodegeneration |  |
|  | DNA damage response of cells |  |  | S phase checkpoint control of lymphoblastoid cell lines | cell death of neuroblastoma cell lines | morphology of cardiovascular system |  |
|  | arrest in interphase of sarcoma cell lines |  |  | X-linked mental retardation type 100 | multi-cancer susceptibility syndrome | abnormal morphology of reproductive system |  |
|  | senescence of tumor cell lines |  |  | abnormal morphology of hepatic vein | quantity of fibroblast cell lines | differentiation of leukocytes |  |
|  | lymphocytic cancer |  |  | abnormal morphology of intrahepatic bile duct | myelomonocytic leukemia | cytostasis of tumor cell lines |  |
|  | exit from mitosis |  |  | abnormal morphology of meiotic configuration | arrest in interphase of tumor cells | development of endocrine gland |  |
|  | mitotic catastrophe |  |  | advanced stage neuroblastoma | patterning of neurons | production of reactive oxygen species |  |
|  | survival of organism |  |  | aneuploidy of colon carcinoma cells | formation of asters | expression of reporter protein |  |
|  | DNA replication checkpoint of cells |  |  | aneuploidy of hepatocytes | re-entry into S phase of bone cancer cell lines | communication of cells |  |
|  | quantity of replication fork |  |  | apoptosis of DN2 cells | re-entry into S phase of sarcoma cell lines | necrosis of liver |  |
|  | arrest in G2 phase of cervical cancer cell lines |  |  | apoptosis of pro-T3 thymocytes | gonadal tumor | abnormal morphology of visceral endoderm |  |
|  | homologous recombination of DNA |  |  | apoptosis of rhabdoid cell lines | malignant neoplasm of heart, mediastinum and pleura | persistent truncus arteriosus |  |
|  | arrest in G2 phase of sarcoma cell lines |  |  | arrest in G0/G1 phase transition of myeloma cell lines | rasopathy | quantity of phagocytes |  |
|  | lung cancer |  |  | arrest in G1/S phase transition of embryonic stem cells | cell death of lymphoid organ | progressive prostate carcinoma |  |
|  | abnormal morphology of Nucleus |  |  | arrest in G2/M phase of cardiomyocytes | pharyngeal cancer | apoptosis of hematopoietic progenitor cells |  |
|  | quantity of microtubules |  |  | arrest in G2/M phase of leukemia cell lines | morphology of cerebral cortex | abnormal morphology of membrane tissue |  |
|  | growth of liver tumor |  |  | arrest in differentiation of neuroblastoma cells | primary neoplasm | differentiation of fibroblast cell lines |  |
|  | S phase checkpoint control of tumor cell lines |  |  | arrest in late prometaphase of cervical cancer cell lines | autophagic cell death | hypertrophy of heart |  |
|  | aneuploidy of epithelial cells |  |  | arrest in metaphase of lung cell lines | quantity of adenoma | cell death of hepatoma cell lines |  |
|  | separation of centrosome |  |  | arrest in mitosis of ovarian cancer cells | stratification of cerebral cortex | quantity of T lymphocytes |  |
|  | initiation of replication of DNA |  |  | arrest in mitosis of prostate cancer cell lines | Ovarian Cancer and Tumors | hypertrophic response of cardiomyocytes |  |
|  | polymerization of microtubules |  |  | arrest in prometaphase of fibroblast cell lines | apoptosis of myeloma cell lines | size of connective tissue cells |  |
|  | delay in initiation of prometaphase |  |  | arrest in spindle checkpoint of cervical cancer cell lines | cell death of stem cells | concentration of acylglycerol |  |
|  | G2 phase of fibroblast cell lines |  |  | assembly of midzone | differentiation of skin | pelvic organ prolapse |  |
|  | tumorigenesis of sarcoma |  |  | assembly of septin ring | apoptosis of leukemia cell lines | cell proliferation of T lymphocytes |  |
|  | survival of sarcoma cell lines |  |  | autophagic cell death of pancreatic cancer cell lines | thymic lymphoma | transport of molecule |  |
|  | aneuploidy of fibroblasts |  |  | autosomal recessive primary microcephaly type 5 | biosynthesis of nucleoside triphosphate | proliferation of prostate cancer cell lines |  |
|  | checkpoint control of mitotic spindle |  |  | autosomal recessive primary microcephaly type 7 | entry into M phase | apoptosis of leukocyte cell lines |  |
|  | homologous recombination of plasmid DNA |  |  | binding of double-stranded DNA | repair of cells | function of muscle |  |
|  | polyploidization of embryonic cell lines |  |  | bleeding of lung tissue | mismatch repair | hypopituitarism |  |
|  | arrest in G1 phase of tumor cell lines |  |  | bridging of chromosomes | stage 4 non-Sezary/visceral disease | formation of lung |  |
|  | G1 phase of tumor cell lines |  |  | caspase-independent cell death of pancreatic cancer cell lines | nasopharyngeal carcinoma | degeneration of cells |  |
|  | aneuploidy of splenocytes |  |  | cell division of centromeres | G2/M phase of tumor cell lines | proliferation of lymphocytes |  |
|  | arrest in interphase of embryonic stem cells |  |  | cell survival of squamous cell carcinoma cell lines | apoptosis of lymphoid organ | apoptosis of prostate cancer cell lines |  |
|  | polyploidization of tumor cell lines |  |  | chromosomal congression of bone cancer cell lines | proliferation of melanocytes | quantity of alveolar epithelium |  |
|  | arrest in cell cycle progression of cervical cancer cell lines |  |  | chromosomal congression of sarcoma cell lines | advanced stage solid tumor | abnormal morphology of dilated seminiferous tubule |  |
|  | female genital tract adenocarcinoma |  |  | cytostasis of mammary tumor cells | invasion of tumor cells | abnormal morphology of lung |  |
|  | chromosomal congression of metaphase plate |  |  | cytostasis of stomach cancer cell lines | cell viability of fibrosarcoma cell lines | advanced androgen-dependent prostate tumor |  |
|  | cell death of bone cancer cell lines |  |  | deformation of spindle pole | delay in tumorigenesis of mammary tumor | atrophic vaginitis |  |
|  | tumorigenesis of non small cell lung adenocarcinoma |  |  | delay in initiation of anaphase of breast cancer cell lines | ploidy of hepatocytes | axillary lymph node negative breast cancer |  |
|  | synthesis of DNA |  |  | delay in initiation of anaphase of fibroblast cell lines | quantity of leukemia cell lines | dyspareunia |  |
|  | tumorigenesis of soft tissue sarcoma |  |  | delay in initiation of formation of liver tumor | size of lung tumor | estrogen receptor positive intraepithelial neoplasia |  |
|  | breakage of chromosomes |  |  | delay in initiation of mitotic exit of cervical cancer cell lines | tumorigenesis of non-Hodgkin lymphoma | estrogen receptor positive invasive breast adenocarcinoma |  |
|  | repair of tumor cell lines |  |  | delay in segregation of sister chromatids | aberration of chromosomes | female hypogonadism |  |
|  | proliferation of pancreatic cancer cell lines |  |  | density of melanoma | arrest in development of organism | fibrocystic disease of breast |  |
|  | delay in M phase of tumor cell lines |  |  | dissociation of embryonic stem cells | large cell transformed mycosis fungoides | function of vagina |  |
|  | cell proliferation of cervical cancer cell lines |  |  | double-stranded DNA break repair of B-lymphocyte derived cell lines | quantity of tumor | hypoestrogenism |  |
|  | apoptosis of sarcoma cell lines |  |  | endoreduplication of cervical cancer cell lines | enteropathy associated T cell lymphoma | hypoplasia of vagina |  |
|  | association of chromatin |  |  | enlargement of pituitary gland | M phase of breast cancer cell lines | locally advanced estrogen receptor positive HER2 negative breast cancer |  |
|  | non-Hodgkin's disease |  |  | entry into S phase of liver cell lines | Proteus mirabilis infection | microscopic hematuria |  |
|  | mitosis of bone cancer cell lines |  |  | entry into cell cycle progression of megakaryocytes | accumulation of embryonic cell lines | postmenopausal vulvar atrophy |  |
|  | mitotic catastrophe of tumor cell lines |  |  | entry into mitosis of pancreatic cancer cell lines | accumulation of kidney cell lines | recurrent urinary tract infection |  |
|  | ploidy of fibroblast cell lines |  |  | entry into senescence of breast cancer cell lines | anaphase of fibroblasts | transformation of Sertoli-like cells |  |
|  | quantity of micronuclei |  |  | exit from M phase of cervical cancer cell lines | arrest in mitosis of fibroblast cell lines | vulvar lichen sclerosus |  |
|  | arrest in G2 phase of carcinoma cell lines |  |  | focal segmental glomerulosclerosis type 8 | association of kinetochores | cell death of T lymphocytes |  |
|  | apoptosis of bone cancer cell lines |  |  | formation of arterial wall | bridging of nucleus | cell transformation |  |
|  | organization of organelle |  |  | formation of macronuclei | inflammation of endothelial cells | abnormal morphology of pericardium |  |
|  |  |  |  | formation of sarcoma cell lines | low grade malignant neoplasm of prostate | cell movement of breast cancer cell lines |  |
|  |  |  |  | grade 1 dysplasia | mitotic catastrophe of cervical cancer cell lines | differentiation of erythroid progenitor cells |  |
|  |  |  |  | grade 3 bladder carcinoma | mutagenesis of lung cells | interphase of breast cancer cell lines |  |
|  |  |  |  | growth of bladder carcinoma | mutagenesis of splenocytes | function of cardiovascular system |  |
|  |  |  |  | growth of rhabdoid cell lines | regulation of neutrophils | fibrosis of liver |  |
|  |  |  |  | hypercondensation of chromatin | single-stranded DNA break repair of tumor cell lines | development of metanephros |  |
|  |  |  |  | hypercondensation of chromosomes | spindle checkpoint of fibroblasts | migration of endothelial cells |  |
|  |  |  |  | hyperplasia of prostate epithelium | stratification of neurons | alopecia areata |  |
|  |  |  |  | hyperplasia of seminal vesicle | survival of cartilage tissue | release of corticosterone |  |
|  |  |  |  | hypertrophy of myometrium | synthesis of CTP | development of mesenchyme |  |
|  |  |  |  | hypodiploidy of prostate cancer cell lines | acidosis | inflammation of body cavity |  |
|  |  |  |  | interkinetic nuclear migration | bone sarcoma | Fibrosis |  |
|  |  |  |  | invasion of PTC cells | quantity of embryo | morphology of reproductive system |  |
|  |  |  |  | loss of bone marrow-derived mononuclear cells | homologous recombination of cells | progression of tumor |  |
|  |  |  |  | mastocytoma | differentiation of epidermal cells | migration of vascular endothelial cells |  |
|  |  |  |  | maturation of peripheral T lymphocyte | delay in M phase of cervical cancer cell lines | congenital heart disease |  |
|  |  |  |  | metaphase of lung cancer cell lines | pigmentation of cells | mass of testis |  |
|  |  |  |  | metastatic esophageal squamous cell cancer | vulva cancer | development of sensory organ |  |
|  |  |  |  | microcephaly with or without chorioretinopathy, lymphedema, or mental retardation | formation of nucleus | formation of embryonic tissue |  |
|  |  |  |  | migratory capacity of lung cancer cell lines | hyperplasia of prostate gland | morphogenesis of cardiovascular system |  |
|  |  |  |  | mirror movements type 2 | G1 phase of fibroblast cell lines | incorporation of leucine |  |
|  |  |  |  | mitosis of endothelial cell lines | viability | size of neuroendocrine cells |  |
|  |  |  |  | mitosis of eye cell lines | incidence of thymic lymphoma | corticosteroid-responsive dermatosis |  |
|  |  |  |  | mitosis of megakaryocytes | tumorigenesis of head and neck tumor | phimosis |  |
|  |  |  |  | mitosis of trophoblast giant cells | tumorigenesis of adenocarcinoma | formation of muscle |  |
|  |  |  |  | mitotic catastrophe of rhabdoid cell lines | instability of chromosomes | differentiation of embryonic tissue |  |
|  |  |  |  | mitotic index of embryonic cell lines | incidence of lung tumor | differentiation of erythroid cells |  |
|  |  |  |  | mitotic index of epithelial cell lines | advanced stage carcinoma | accumulation of lipid |  |
|  |  |  |  | mitotic index of kidney cell lines | hydrolysis of ATP | development of internal genitalia |  |
|  |  |  |  | mosaic variegated aneuploidy syndrome type 1 | entry into S phase of muscle cells | differentiation of regulatory T lymphocytes |  |
|  |  |  |  | natural killer cell and glucocorticoid deficiency with DNA repair defect | blood protein disorder | neurogenesis of brain |  |
|  |  |  |  | onset of mitosis of embryonic stem cells | quantity of embryonic tissue | maturation of cells |  |
|  |  |  |  | onset of tumorigenesis of skin | phosphorylation of L-threonine | synthesis of steroid hormone |  |
|  |  |  |  | organization of DNA | transformation of embryonic cell lines | atresia |  |
|  |  |  |  | outgrowth of embryoblast | incidence of sarcoma | efficacy of beta-estradiol |  |
|  |  |  |  | polyploidization of epithelial cell lines |  | primary estrogen receptor positive breast cancer |  |
|  |  |  |  | polyploidization of kidney cell lines |  | transformation of granulosa cells |  |
|  |  |  |  | polyploidy of cardiomyocytes |  | uterine prolapse |  |
|  |  |  |  | polyploidy of liver cell lines |  | memory |  |
|  |  |  |  | progressive heart failure |  | development of gastrointestinal tract |  |
|  |  |  |  | proliferation of Clara cells |  | quantity of osteoblasts |  |
|  |  |  |  | prophase of lung cancer cell lines |  | morphology of vessel |  |
|  |  |  |  | quantity of apoptotic colorectal cancer cell lines |  | activation of cells |  |
|  |  |  |  | quantity of macrocytes |  | repression of RNA |  |
|  |  |  |  | reduplication of centrosome |  | apoptosis of cancer cells |  |
|  |  |  |  | sensitivity of epithelial cell lines |  | dysplasia |  |
|  |  |  |  | sister chromatid exchange of DNA |  | familial cardiac septal defect |  |
|  |  |  |  | size of pulmonary adenoma |  | ulcerative proctitis |  |
|  |  |  |  | sphere formation of cervical cancer cell lines |  | quantity of red blood cells |  |
|  |  |  |  | sphere formation of neural stem cells |  | vasodilation of artery |  |
|  |  |  |  | structural integrity of asters |  | development of neural crest cells |  |
|  |  |  |  | survival of Bloom's Syndrome lymphoblastoid cell lines |  | glucose tolerance |  |
|  |  |  |  | survival of rhabdoid cell lines |  | formation of pathological cyst |  |
|  |  |  |  | transition of telencephalic progenitor cells |  | differentiation of embryonic stem cells |  |
|  |  |  |  | tumorigenesis of bladder tissue |  | morphology of endocrine gland |  |
|  |  |  |  | bundling of microtubules |  | size of adipocytes |  |
|  |  |  |  | Pharyngeal Cancer and Tumors |  | hypoplasia of thorax |  |
|  |  |  |  | necrosis of brain cancer cell lines |  | quantity of mineral |  |
|  |  |  |  | carcinoma in breast |  | secretion of lipid |  |
|  |  |  |  |  |  | area of compact bone |  |
|  |  |  |  |  |  | metastatic prostate carcinoma |  |
|  |  |  |  |  |  | apoptosis of T lymphocytes |  |
|  |  |  |  |  |  | function of uterus |  |
|  |  |  |  |  |  | metabolism of hormone |  |
|  |  |  |  |  |  | development of cardiovascular tissue |  |
|  |  |  |  |  |  | apoptosis of hepatoma cell lines |  |
|  |  |  |  |  |  | osteoporosis |  |
|  |  |  |  |  |  | experimental autoimmune encephalomyelitis |  |
|  |  |  |  |  |  | apoptosis of fibroblast cell lines |  |
|  |  |  |  |  |  | growth of genital organ |  |
|  |  |  |  |  |  | mass of genitourinary system |  |
|  |  |  |  |  |  | abnormal morphology of ear |  |
|  |  |  |  |  |  | apoptosis of B-lymphocyte derived cell lines |  |
|  |  |  |  |  |  | hepatomegaly |  |
|  |  |  |  |  |  | quantity of lymphatic system component |  |
|  |  |  |  |  |  | abnormal morphology of heart |  |
|  |  |  |  |  |  | abnormal morphology of long femur |  |
|  |  |  |  |  |  | intromission |  |
|  |  |  |  |  |  | metastatic HER2 non-overexpressing hormone receptor positive breast cancer |  |
|  |  |  |  |  |  | stage 1 progesterone receptor-positive breast cancer |  |
|  |  |  |  |  |  | stage 2 progesterone receptor-positive breast cancer |  |
|  |  |  |  |  |  | stage 3 estrogen receptor positive malignant neoplasm of breast |  |
|  |  |  |  |  |  | stage 3 progesterone receptor-positive breast cancer |  |
|  |  |  |  |  |  | postmenopausal osteoporosis |  |
|  |  |  |  |  |  | proliferation of Sertoli cells |  |
|  |  |  |  |  |  | circadian rhythm |  |
|  |  |  |  |  |  | arrest in proliferation of tumor cell lines |  |
|  |  |  |  |  |  | Organ Degeneration |  |
|  |  |  |  |  |  | area of cells |  |
|  |  |  |  |  |  | hyperplasia of epithelial tissue |  |
|  |  |  |  |  |  | binding of PPAR response element |  |
|  |  |  |  |  |  | accumulation of bile acid |  |
|  |  |  |  |  |  | adipogenesis of epididymal fat |  |
|  |  |  |  |  |  | berylliosis |  |
|  |  |  |  |  |  | erythroblastopenia anemia |  |
|  |  |  |  |  |  | neoplasia of forestomach |  |
|  |  |  |  |  |  | post-traumatic arthritis |  |
|  |  |  |  |  |  | quantity of ursodeoxycholic acid |  |
|  |  |  |  |  |  | viral pneumonia |  |
|  |  |  |  |  |  | adipogenesis of adipose tissue |  |
|  |  |  |  |  |  | hypoplasia of uterus |  |
|  |  |  |  |  |  | abnormal morphology of cardiovascular system |  |
|  |  |  |  |  |  | paralysis |  |
|  |  |  |  |  |  | failure of heart |  |
|  |  |  |  |  |  | apoptosis of leukocytes |  |
|  |  |  |  |  |  | concentration of D-glucose |  |
|  |  |  |  |  |  | size of endocrine gland |  |
|  |  |  |  |  |  | differentiation of oligodendrocytes |  |
|  |  |  |  |  |  | damage of bone |  |
|  |  |  |  |  |  | volume of tumor |  |
|  |  |  |  |  |  | chronic pulmonary disease |  |
|  |  |  |  |  |  | atherosclerosis |  |
|  |  |  |  |  |  | damage of liver |  |
|  |  |  |  |  |  | quantity of lymphoid organ |  |
|  |  |  |  |  |  | hyperplasia of blood cells |  |
|  |  |  |  |  |  | Hypertension |  |
|  |  |  |  |  |  | abnormal morphology of epithelial tissue |  |
|  |  |  |  |  |  | hypoplasia of muscle |  |
|  |  |  |  |  |  | insulin sensitivity index |  |
|  |  |  |  |  |  | development of striated muscle |  |
|  |  |  |  |  |  | neurogenesis of hippocampus |  |
|  |  |  |  |  |  | adrenocorticotropic hormone (ACTH) deficiency |  |
|  |  |  |  |  |  | anoikis of RPE cells |  |
|  |  |  |  |  |  | metastatic malignant neoplasm of prostate |  |
|  |  |  |  |  |  | morphology of epithelial cells |  |
|  |  |  |  |  |  | quantity of antigen presenting cells |  |
|  |  |  |  |  |  | apoptosis of endothelial cells |  |
|  |  |  |  |  |  | quantity of central nervous system cells |  |
|  |  |  |  |  |  | Dermatitis |  |
|  |  |  |  |  |  | ulcerative colitis |  |
|  |  |  |  |  |  | breast or ovarian carcinoma |  |
|  |  |  |  |  |  | body mass index |  |
|  |  |  |  |  |  | motor function |  |
|  |  |  |  |  |  | morphology of gland |  |
|  |  |  |  |  |  | size of heart |  |
|  |  |  |  |  |  | progression of prostatic tumor |  |
|  |  |  |  |  |  | contractility of muscle |  |
|  |  |  |  |  |  | proliferation of epithelial cell lines |  |
|  |  |  |  |  |  | abnormal morphology of myocardium |  |
|  |  |  |  |  |  | differentiation of lymphocytes |  |
|  |  |  |  |  |  | invasive breast adenocarcinoma |  |
|  |  |  |  |  |  | benign neoplasm of endocrine gland |  |
|  |  |  |  |  |  | ossification of bone |  |
|  |  |  |  |  |  | Rheumatic Disease |  |
|  |  |  |  |  |  | infertility |  |
|  |  |  |  |  |  | differentiation of phagocytes |  |
|  |  |  |  |  |  | neuroendocrine tumor |  |
|  |  |  |  |  |  | response of heart |  |
|  |  |  |  |  |  | quantity of macrophages |  |
|  |  |  |  |  |  | maturation of reproductive tract |  |
|  |  |  |  |  |  | differentiation of stem cells |  |
|  |  |  |  |  |  | development of epithelial tissue |  |
|  |  |  |  |  |  | G1 phase of breast cancer cell lines |  |
|  |  |  |  |  |  | size of tumor |  |
|  |  |  |  |  |  | hyperplasia of epithelial cells |  |
|  |  |  |  |  |  | abnormal morphology of labyrinthine zone of placenta |  |
|  |  |  |  |  |  | neurocristopathy syndrome |  |
|  |  |  |  |  |  | abnormal morphology of ovary |  |
|  |  |  |  |  |  | hypertrophy of tissue |  |
|  |  |  |  |  |  | cell death of phagocytes |  |
|  |  |  |  |  |  | disorder of pregnancy |  |
|  |  |  |  |  |  | mass of retroperitoneal fat pad |  |
|  |  |  |  |  |  | differentiation of mononuclear leukocytes |  |
|  |  |  |  |  |  | myocardial dysfunction |  |
|  |  |  |  |  |  | concentration of testosterone |  |
|  |  |  |  |  |  | urothelial cancer |  |
|  |  |  |  |  |  | release of nitric oxide |  |
|  |  |  |  |  |  | morphology of blood vessel |  |
|  |  |  |  |  |  | apoptosis of erythroid cell lines |  |
|  |  |  |  |  |  | binding of mouse mammary tumor virus |  |
|  |  |  |  |  |  | development of lymphatic system |  |
|  |  |  |  |  |  | differentiation of T lymphocytes |  |
|  |  |  |  |  |  | osteopenia |  |
|  |  |  |  |  |  | blood pressure |  |
|  |  |  |  |  |  | morphology of connective tissue cells |  |
|  |  |  |  |  |  | concentration of progesterone |  |
|  |  |  |  |  |  | obesity |  |
|  |  |  |  |  |  | mass of genital organ |  |
|  |  |  |  |  |  | burn |  |
|  |  |  |  |  |  | arrest in cell cycle progression of neuronal hybrid cells |  |
|  |  |  |  |  |  | differentiation of neuronal hybrid cells |  |
|  |  |  |  |  |  | growth of atretic ovarian follicle |  |
|  |  |  |  |  |  | steroidogenesis of hormone |  |
|  |  |  |  |  |  | hepatic steatosis |  |
|  |  |  |  |  |  | tauopathy |  |
|  |  |  |  |  |  | length of neurites |  |
|  |  |  |  |  |  | oxidative stress |  |
|  |  |  |  |  |  | development of lymphocytes |  |
|  |  |  |  |  |  | anoikis of cervical cancer cell lines |  |
|  |  |  |  |  |  | function of enterocytes |  |
|  |  |  |  |  |  | quantity of desmosomes |  |
|  |  |  |  |  |  | urination disorder |  |
|  |  |  |  |  |  | regeneration of epithelial tissue |  |
|  |  |  |  |  |  | quantity of FSH |  |
|  |  |  |  |  |  | size of secretory structure |  |
|  |  |  |  |  |  | response of muscle cells |  |
|  |  |  |  |  |  | neurogenesis of nervous system |  |
|  |  |  |  |  |  | morphology of heart |  |
|  |  |  |  |  |  | ovulation |  |
|  |  |  |  |  |  | proliferation of gonadal cells |  |
|  |  |  |  |  |  | preterm birth or low birth weight |  |
|  |  |  |  |  |  | function of genital organ |  |
|  |  |  |  |  |  | binding of gene |  |
|  |  |  |  |  |  | quantity of carbohydrate |  |
|  |  |  |  |  |  | morphology of cardiac muscle |  |
|  |  |  |  |  |  | learning |  |
|  |  |  |  |  |  | hypogonadism |  |
|  |  |  |  |  |  | mass of reproductive tract |  |
|  |  |  |  |  |  | familial congenital heart disease |  |
|  |  |  |  |  |  | secretion of steroid |  |
|  |  |  |  |  |  | cellular infiltration |  |
|  |  |  |  |  |  | abnormal morphology of blood vessel |  |
|  |  |  |  |  |  | proliferation of liver cells |  |
|  |  |  |  |  |  | abnormal morphology of liver |  |
|  |  |  |  |  |  | arrest in G0/G1 phase transition |  |
|  |  |  |  |  |  | concentration of ATP |  |
|  |  |  |  |  |  | quantity of brain cells |  |
|  |  |  |  |  |  | secretion of hormone |  |
|  |  |  |  |  |  | development of endothelial tissue |  |
|  |  |  |  |  |  | colony formation of tumor cell lines |  |
|  |  |  |  |  |  | loss of dopaminergic neurons |  |
|  |  |  |  |  |  | mass of fat |  |
|  |  |  |  |  |  | morphology of gonad |  |
|  |  |  |  |  |  | inflammation of liver |  |
|  |  |  |  |  |  | hypertrophy of cells |  |
|  |  |  |  |  |  | fatty acid metabolism |  |
|  |  |  |  |  |  | maturation of ovarian follicle |  |
|  |  |  |  |  |  | export of molecule |  |
|  |  |  |  |  |  | concentration of bile acid |  |
|  |  |  |  |  |  | energy expenditure |  |
|  |  |  |  |  |  | homeostasis of D-glucose |  |
|  |  |  |  |  |  | size of body |  |
|  |  |  |  |  |  | insulin sensitivity |  |

**Pathways overlap**

| **5 common elements in "RB1", "LOH" and "E2F":** | **13 common elements in "RB1" and "LOH":** | **8 common elements in "LOH" and "E2F":** | **26 elements included exclusively in "LOH":** | **100 elements included exclusively in "E2F":** |
| --- | --- | --- | --- | --- |
| ATM Signaling | Mitotic Roles of Polo-Like Kinase | Molecular Mechanisms of Cancer | Regulation of Cellular Mechanics by Calpain Protease | Hypoxia Signaling in the Cardiovascular System |
| Estrogen-mediated S-phase Entry | Cell Cycle Control of Chromosomal Replication | Non-Small Cell Lung Cancer Signaling | Cell Cycle: G1/S Checkpoint Regulation | eNOS Signaling |
| Aryl Hydrocarbon Receptor Signaling | Cell Cycle: G2/M DNA Damage Checkpoint Regulation | Glioblastoma Multiforme Signaling | Protein Ubiquitination Pathway | Protein Kinase A Signaling |
| Pancreatic Adenocarcinoma Signaling | Role of CHK Proteins in Cell Cycle Checkpoint Control | Adipogenesis pathway | Ovarian Cancer Signaling | AMPK Signaling |
| Antiproliferative Role of TOB in T Cell Signaling | Role of BRCA1 in DNA Damage Response | Chronic Myeloid Leukemia Signaling | PCP pathway | Role of Oct4 in Mammalian Embryonic Stem Cell Pluripotency |
|  | Cyclins and Cell Cycle Regulation | GDNF Family Ligand-Receptor Interactions | Granzyme B Signaling | Glucocorticoid Receptor Signaling |
|  | Hereditary Breast Cancer Signaling | Sertoli Cell-Sertoli Cell Junction Signaling | Melanoma Signaling | PXR/RXR Activation |
|  | Pyridoxal 5'-phosphate Salvage Pathway | NRF2-mediated Oxidative Stress Response | Glioma Signaling | FXR/RXR Activation |
|  | Salvage Pathways of Pyrimidine Ribonucleotides |  | Telomerase Signaling | PPAR Signaling |
|  | p53 Signaling |  | Cholesterol Biosynthesis I | BMP signaling pathway |
|  | Mismatch Repair in Eukaryotes |  | Cholesterol Biosynthesis II (via 24,25-dihydrolanosterol) | Estrogen-Dependent Breast Cancer Signaling |
|  | DNA damage-induced 14-3-3Ïƒ Signaling |  | Cholesterol Biosynthesis III (via Desmosterol) | RAR Activation |
|  | Pyrimidine Deoxyribonucleotides De Novo Biosynthesis I |  | Breast Cancer Regulation by Stathmin1 | TGF-Î² Signaling |
|  |  |  | Cell Cycle Regulation by BTG Family Proteins | Hepatic Cholestasis |
|  |  |  | DNA Methylation and Transcriptional Repression Signaling | Thyroid Cancer Signaling |
|  |  |  | Superpathway of Cholesterol Biosynthesis | ERK/MAPK Signaling |
|  |  |  | Myc Mediated Apoptosis Signaling | Transcriptional Regulatory Network in Embryonic Stem Cells |
|  |  |  | Glutamate Removal from Folates | Corticotropin Releasing Hormone Signaling |
|  |  |  | Role of IL-17A in Psoriasis | Estrogen Receptor Signaling |
|  |  |  | Tight Junction Signaling | Role of IL-17F in Allergic Inflammatory Airway Diseases |
|  |  |  | Vitamin-C Transport | PI3K Signaling in B Lymphocytes |
|  |  |  | RAN Signaling | Neurotrophin/TRK Signaling |
|  |  |  | Epoxysqualene Biosynthesis | PPARÎ±/RXRÎ± Activation |
|  |  |  | Guanine and Guanosine Salvage I | Prostate Cancer Signaling |
|  |  |  | L-glutamine Biosynthesis II (tRNA-dependent) | LPS-stimulated MAPK Signaling |
|  |  |  | GADD45 Signaling | ERK5 Signaling |
|  |  |  |  | FLT3 Signaling in Hematopoietic Progenitor Cells |
|  |  |  |  | Oncostatin M Signaling |
|  |  |  |  | Mouse Embryonic Stem Cell Pluripotency |
|  |  |  |  | Thrombin Signaling |
|  |  |  |  | Melanocyte Development and Pigmentation Signaling |
|  |  |  |  | Cardiomyocyte Differentiation via BMP Receptors |
|  |  |  |  | FGF Signaling |
|  |  |  |  | Role of NANOG in Mammalian Embryonic Stem Cell Pluripotency |
|  |  |  |  | NGF Signaling |
|  |  |  |  | 4-1BB Signaling in T Lymphocytes |
|  |  |  |  | LPS/IL-1 Mediated Inhibition of RXR Function |
|  |  |  |  | GNRH Signaling |
|  |  |  |  | Synaptic Long Term Potentiation |
|  |  |  |  | GÎ±s Signaling |
|  |  |  |  | P2Y Purigenic Receptor Signaling Pathway |
|  |  |  |  | Circadian Rhythm Signaling |
|  |  |  |  | MIF-mediated Glucocorticoid Regulation |
|  |  |  |  | Unfolded protein response |
|  |  |  |  | Role of IL-17A in Arthritis |
|  |  |  |  | Xenobiotic Metabolism Signaling |
|  |  |  |  | Glioma Invasiveness Signaling |
|  |  |  |  | IL-12 Signaling and Production in Macrophages |
|  |  |  |  | Endometrial Cancer Signaling |
|  |  |  |  | Regulation of the Epithelial-Mesenchymal Transition Pathway |
|  |  |  |  | Wnt/Ca+ pathway |
|  |  |  |  | Nur77 Signaling in T Lymphocytes |
|  |  |  |  | Role of MAPK Signaling in the Pathogenesis of Influenza |
|  |  |  |  | PEDF Signaling |
|  |  |  |  | IL-17 Signaling |
|  |  |  |  | CREB Signaling in Neurons |
|  |  |  |  | B Cell Receptor Signaling |
|  |  |  |  | Ephrin Receptor Signaling |
|  |  |  |  | Dendritic Cell Maturation |
|  |  |  |  | Calcium Signaling |
|  |  |  |  | Factors Promoting Cardiogenesis in Vertebrates |
|  |  |  |  | Regulation of IL-2 Expression in Activated and Anergic T Lymphocytes |
|  |  |  |  | ILK Signaling |
|  |  |  |  | Melatonin Signaling |
|  |  |  |  | Cholecystokinin/Gastrin-mediated Signaling |
|  |  |  |  | Huntington's Disease Signaling |
|  |  |  |  | Acyl-CoA Hydrolysis |
|  |  |  |  | HGF Signaling |
|  |  |  |  | Prolactin Signaling |
|  |  |  |  | Renin-Angiotensin Signaling |
|  |  |  |  | cAMP-mediated signaling |
|  |  |  |  | VDR/RXR Activation |
|  |  |  |  | IGF-1 Signaling |
|  |  |  |  | Cardiac Hypertrophy Signaling |
|  |  |  |  | Neuropathic Pain Signaling In Dorsal Horn Neurons |
|  |  |  |  | TR/RXR Activation |
|  |  |  |  | Phospholipase C Signaling |
|  |  |  |  | Parkinson's Signaling |
|  |  |  |  | Polyamine Regulation in Colon Cancer |
|  |  |  |  | VEGF Signaling |
|  |  |  |  | Assembly of RNA Polymerase III Complex |
|  |  |  |  | G-Protein Coupled Receptor Signaling |
|  |  |  |  | RhoGDI Signaling |
|  |  |  |  | Type II Diabetes Mellitus Signaling |
|  |  |  |  | p38 MAPK Signaling |
|  |  |  |  | PTEN Signaling |
|  |  |  |  | Role of Macrophages, Fibroblasts and Endothelial Cells in Rheumatoid Arthritis |
|  |  |  |  | IL-22 Signaling |
|  |  |  |  | LXR/RXR Activation |
|  |  |  |  | PI3K/AKT Signaling |
|  |  |  |  | IL-17A Signaling in Gastric Cells |
|  |  |  |  | Role of JAK family kinases in IL-6-type Cytokine Signaling |
|  |  |  |  | Role of Pattern Recognition Receptors in Recognition of Bacteria and Viruses |
|  |  |  |  | Insulin Receptor Signaling |
|  |  |  |  | Relaxin Signaling |
|  |  |  |  | Cdc42 Signaling |
|  |  |  |  | Acute Phase Response Signaling |
|  |  |  |  | Maturity Onset Diabetes of Young (MODY) Signaling |
|  |  |  |  | Dopamine-DARPP32 Feedback in cAMP Signaling |
|  |  |  |  | Role of NFAT in Regulation of the Immune Response |
